# Supplementary material for: The effects of co-designed physical activity interventions in older adults: A systematic review and meta-analysis
Source: PLoS One. 2024 May 10;19(5):e0297675. doi: 10.1371/journal.pone.0297675 (PMC11086838; doi:10.1371/journal.pone.0297675)
Supplement: S1 File — (DOCX) [file pone.0297675.s001.docx]

S1 File. Search strategy.

**Final search strategies for MEDLINE, AgeLine, CINAHL, EMBASE, and SPORTDiscus electronic databases.**

The search strategy for MEDLINE, from which the other search strategies were adapted, was reviewed and approved by our supervisors as well as Rachel Couban, a librarian at the McMaster Health Sciences Library.

**MEDLINE (Ovid, 1946 to Present)**

Search conducted: February 28, 2022

Limits: No limits were applied to this search strategy

Number of studies retrieved: 8395

1       exp Exercise/ (226514)

2       exp Exercise Therapy/ (58515)

3       exp Recreation/ (230754)

4       exp Recreation Therapy/ (134)

5       exp Motor Activity/ (324691)

6       exp Movement/ (620672)

7       exp Sports/ (202767)

8       exp Sports Equipment/ (1572)

9       exp "Activities of Daily Living"/ (113463)

10     exp Physical Fitness/ (34372)

11     exp Walking/ (62096)

12     exp Exercise Movement Techniques/ (9524)

13     exp Physical Endurance/ (36079)

14     abdominal exercis*.mp. (108)

15     activit* of daily living.mp. (84984)

16     aerobic exercis*.mp. (11551)

17     anaerobic exercis*.mp. (656)

18     arm exercis*.mp. (547)

19     aquatic exercis*.mp. (451)

20     breathing exercis*.mp. (4489)

21     cardiorespiratory fitness.mp. (7176)

22     closed kinetic chain exercis*.mp. (150)

23     danc*.mp. (9302)

24     dynamic exercis*.mp. (2174)

25     exercis*.mp. (429251)

26 (exercise and fitness equipment).mp. (37)

27     exercis* position.mp. (27)

28     exercis* intensity.mp. (5758)

29     exercis* therapy.mp. (47731)

30     fitness.mp. (103702)

31     group exercis*.mp. (1681)

32     isokinetic exercis*.mp. (295)

33     isotonic exercis*.mp. (247)

34     isometric exercis*.mp. (1995)

35     kegel exercis*.mp. (144)

36     leg exercis*.mp. (899)

37     moderate to vigorous physical activ*.mp. (5187)

38     motor activit*.mp. (110105)

39     movement techniqu*.mp. (10404)

40     movement*.mp. (564427)

41     muscle exercis*.mp. (1236)

42     open kinetic chain exercis*.mp. (60)

43     physical fit*.mp. (34130)

44     physical activ*.mp. (134883)

45     plyometric*.mp. (1462)

46     recreation*.mp. (36904)

47     resistance training.mp. (16005)

48     sport*.mp. (119734)

49     sport* equipment.mp. (1751)

50     static exercis*.mp. (465)

51     stretching exercis*.mp. (2926)

52     swim*.mp. (49204)

53     treadmill exercis*.mp. (8148)

54     therapeutic exercis*.mp. (1683)

55     walk*.mp. (144000)

56     warm-up exercis*.mp. (613)

57     1 or 2 or 3 or 4 or 5 or 6 or 7 or 8 or 9 or 10 or 11 or 12 or 13 or 14 or 15 or 16 or 17 or 18 or 19 or 20 or 21 or 22 or 23 or 24 or 25 or 26 or 27 or 28 or 29 or 30 or 31 or 32 or 33 or 34 or 35 or 36 or 37 or 38 or 39 or 40 or 41 or 42 or 43 or 44 or 45 or 46 or 47 or 48 or 49 or 50 or 51 or 52 or 53 or 54 or 55 or 56 (1620405)

58     exp Community-Based Participatory Research/ (5484)

59     exp Patient Participation/ (28271)

60     exp Community Participation/ (46208)

61     action research.mp. (4976)

62     advisory.mp. (25976)

63     co-creat*.mp. (1397)

64     cocreat*.mp. (291)

65     co-design.mp. (1295)

66     codesign.mp. (248)

67     co-produc*.mp. (4231)

68     coproduc*.mp. (1790)

69     co-evaluat*.mp. (80)

70     coevaluat*.mp. (16)

71     co-decision*.mp. (26)

72     codecision*.mp. (4)

73     co-method*.mp. (70)

74     community-based participatory research.mp. (6923)

75     community participation.mp. (20748)

76     engagement.mp. (84358)

77     participatory design.mp. (715)

78     participatory research.mp. (8071)

79     participatory health research.mp. (88)

80     patient participat*.mp. (30372)

81     client participat*.mp. (136)

82     (public and patient involvement).mp. (494)

83     user involvement.mp. (1051)

84     58 or 59 or 60 or 61 or 62 or 63 or 64 or 65 or 66 or 67 or 68 or 69 or 70 or 71 or 72 or 73 or 74 or 75 or 76 or 77 or 78 or 79 or 80 or 81 or 82 or 83 (174185)

85     exp Aged/ (3374713)

86     exp Aging/ (281957)

87     aged.mp. (5791556)

88     ageing.mp. (50032)

89     aged individual*.mp. (2752)

90     aged hospital patient*.mp. (13)

91     aged patient*.mp. (6622)

92     aged client*.mp. (28)

93     aged worker*.mp. (172)

94     age 60.mp. (12551)

95     age 65.mp. (16359)

96     age 70.mp. (8042)

97     age 75.mp. (5611)

98     age 80.mp. (3509)

99     aging adult*.mp. (822)

100 ageing adult*.mp. (90)

101 elder*.mp. (297575)

102 frail elder*.mp. (15538)

103   geriatric*.mp. (113015)

104   late life.mp. (7904)

105   master* athlete*.mp. (513)

106   old* adult*.mp. (104329)

107   old* patient*.mp. (82757)

108   old* client*.mp. (326)

109   old* people*.mp. (39525)

110   old* veteran*.mp. (730)

111   senior*.mp. (46842)

112   very elderl*.mp. (2512)

113   60 and over.mp. (16172)

114   85 or 86 or 87 or 88 or 89 or 90 or 91 or 92 or 93 or 94 or 95 or 96 or 97 or 98 or 99 or 100 or 101 or 102 or 103 or 104 or 105 or 106 or 107 or 108 or 109 or 110 or 111 or 112 or 113 (6123874)

115   57 and 84 and 114 (8395)

**AgeLine (EBSCOhost, 1978 to Present)**

Search conducted: February 28, 2022

Limits: No limits were applied to this search strategy

Number of studies retrieved: 1169

S1 DE "Exercise" OR DE "Aerobic Exercise" OR DE "Aquatic Exercise" OR DE "Exercise Programs"

S2 DE “Recreation’ OR DE “Recreation Programs”

S3 “Exercise Therapy”

S4 DE “Recreational Therapy”

S5 “Motor Activity”

S6 DE "Movement Therapy" OR DE "Therapy" OR DE "Aromatherapy" OR DE "Art Therapy" OR DE "Behavior Modification" OR DE "Dance Therapy" OR DE "Drama Therapy" OR DE "Drug Therapy" OR DE "Family Therapy" OR DE "Hormone Replacement Therapy" OR DE "Horticultural Therapy" OR DE "Light Therapy" OR DE "Massage" OR DE "Movement Therapy" OR DE "Music Therapy" OR DE "Occupational Therapy" OR DE "Pet Therapy" OR DE "Physical Therapy" OR DE "Psychotherapy" OR DE "Reality Orientation" OR DE "Recreational Therapy" OR DE "Relaxation Therapy" OR DE "Shock Therapy" OR DE "Speech Therapy" OR DE "Validation Therapy"

S7 DE “Sports” OR DE "Physical Activity" OR DE "Bicycling" OR DE "Exercise" OR DE "Running" OR DE "Sports" OR DE "Swimming" OR DE "Walking"

S8 DE “Activities of Daily Living”

S9 DE “Physical Condition” OR DE “Disease Susceptibility” OR DE “Flexibility” OR DE “Recovery”

S10 DE “Walking”

S11 DE “Exercise Programs”

S12 “Physical Endurance”

S13 “abdominal exercis*”

S14 “abdominal exercise”

S15 “activit* of daily living”

S16 “aerobic exercis*”

S17 “anaerobic exercis*”

S18 “arm exercis*”

S19 “aquatic exercis*”

S20 “breathing exercis*”

S21 “cardiorespiratory fitness’

S22 “closed kinetic chain exercis*”

S23 “danc*”

S24 “dynamic exercis*”

S25 “exercis*”

S26 (exercise and fitness equipment)

S27 “exercis* position”

S28 “exercise position”

S29 “exercis* intensity”

S30 “exercis* therapy”

S31 “fitness”

S32 “group exercis*”

S33 “isokinetic exercis*”

S34 “isotonic exercis*”

S35 “isometric exercis*”

S36 “kegel exercis*”

S37 “leg exercis*”

S38 “moderate to vigorous physical activ*”

S39 “motor activ*”

S40 “movement technique*”

S41 “movement*”

S42 “muscle exercis*”

S43 “open kinetic chain exercis*”

S44 “physical fit*”

S45 “physical activ*”

S46 “plyometric*”

S48 “recreation*”

S49 “resistance training”

S50 “sport*”

S51 “sport* equipment”

S52 “static exercis*”

S53 “static exercise”

S54 “stretching exercis*”

S55 “swim*”

S56 “treadmill exercis*”

S57 “therapeutic exercis*”

S58 “walk*”

S59 “warm-up exercis*”

S60 S1ORS2ORS3ORS4 ORS5ORS6ORS7OR S8ORS9ORS10OR S11 ORS12ORS13OR S14 OR S15 OR S16 OR S17 OR S18 OR S19 OR S20 OR S21 OR S22 OR S23 OR S24 OR S25 OR S26 OR S27 OR S28 OR S29 OR S30 OR S31 OR S32 OR S33 OR S34 OR S35 OR S36 OR S37 OR S38 OR S39 OR S40 OR S41 OR S42 OR S43 OR S44 OR S45 OR S46 OR S47 OR S48 OR S49 OR S50 OR S51 OR S52 OR S53 OR S54 OR S55 OR S56 OR S57 OR S58 OR S59

S61 “action research”

S62 “advisory”

S63 “co-creat*”

S64 “cocreat*”

S65 “co-design”

S66 “codesign”

S67 “co-produc*”

S68 “coproduc*”

S69 “co-evaluat*”

S70 “co-evaluate*”

S71 “coevaluat*”

S72 “co-decision*”

S73 “codecision*”

S74 “co-method*”

S75 “community-based participatory research”

S76 “community participation”

S77 “engagement”

S78 “participatory design”

S79 “participatory research”

S80 “participatory health research”

S81 “patient participat*”

S82 “client participat*”

S83 “user involvement”

S84 (public and patient involvement)

S85 S61 OR S62 OR S63 OR S64 OR S65 OR S66 OR S67 OR S68 OR S69 OR S70 OR S71 OR S72 OR S73 OR S74 OR S75 OR S76 OR S77 OR S78 OR S79 OR S80 OR S81 OR S82 OR S83 OR S84

S86 DE "Aging" OR DE "Active Aging" OR DE "Aging in Place" OR DE "Biological Aging" OR DE "Healthy Aging" OR DE "Normal Aging" OR DE "Premature Aging" OR DE "Productive Aging" OR DE "Psychological Aging" OR DE "Successful Aging"

S87 “aged”

S88 “ageing”

S89 “aged individual”

S90 “aged hospital patient”

S91 “aged patient*”

S92 “aged client*”

S93 “aged worker*”

S94 “age 60”

S95 “age 65”

S96 “age 70”

S97 “age 75”

S98 “age 80”

S99 “aging adult*”

S100 “ageing adult*”

S101 “elder*”

S102 “frail elder*”

S103 “geriatric*”

S104 “late life”

S105 “master* athlete*”

S106 “old* adult*”

S107 “old* patient*”

S108 “old* client*”

S109 “old* people*”

S110 “old* veteran*”

S111 “senior*”

S112 “very elderl*”

S113 “60 and over”

S114 S86 OR S87 OR S88 OR S89 OR S90 OR S91 OR S92 OR S93 OR S94 OR S95OR S96 OR S97 OR S98 OR S99 OR S100 OR S101 OR S102 OR S103 OR S104 OR S105 OR S106 OR S107 OR S108 OR S109 OR S110 OR S111 OR S112 OR S113

S115 S60 AND S85 AND S114

**CINAHL** **(EBSCOhost, 1981 to Present)**

Search conducted: February 28, 2022

Limits: No limits were applied to this search strategy

Number of studies retrieved: 3535

S1 MH “Exercise+”

S2 MH “Therapeutic Exercise+”

S3 MH “Recreation+”

S4 MH “Recreational Therapy”

S5 MH “Motor Activity+”

S6 MH “Movement+”

S7 MH “Sports+”

S8 MH “Sports Equipment and Supplies+”

S9 MH “Activities of Daily Living+”

S10 MH “Physical Fitness+”

S11 MH “Walking+”

S12 “exercise movement techniques”

S13 MH “Physical Endurance+”

S14 “abdominal exercis*”

S15 “activit* of daily living”

S16 “aerobic exercis*”

S17 “anaerobic exercis*”

S18 “arm exercis*”

S19 “aquatic exercis*”

S20 “breathing exercis*”

S21 “cardiorespiratory fitness”

S22 “closed kinetic chain exercis*”

S23 “danc*”

S24 “dynamic exercis*”

S25 “exercis*”

S26 (exercise and fitness equipment)

S27 “exercis* position”

S28 “exercis* intensity”

S29 “exercis* therapy”

S30 “fitness”

S31 “group exercis*”

S32 “isokinetic exercis*”

S33 “isotonic exercis*”

S34 “isometric exercis*”

S35 “kegal exercis*”

S36 “leg exercis*”

S37 “moderate to vigorous physical activ*”

S38 “motor activit*”

S39 “movement techniqu*”

S40 “movement*”

S41 “muscle exercis*”

S42 “open kinetic chain exercis*”

S43 “physical fit*”

S44 “physical activ*”

S45 “plyometric*”

S46 “recreation*”

S47 “resistance training”

S48 “sport*”

S49 “sport* equipment”

S50 “static exercis*”

S51 “stretching exercis*”

S52 “swim*”

S53 “treadmill exercis*”

S54 “therapeutic exercis*”

S55 “walk*”

S56 “warm-up exercis*”

S57 S1 OR S2 OR S3 OR S4 OR S5 OR S6 OR S7 OR S8 OR S9 OR S10 OR S11 OR S12 OR S13 OR S14 OR S15 OR S16 OR S17 OR S18 OR S19 OR S20 OR S21 OR S22 OR S23 OR S24 OR S25 OR S26 OR S27 OR S28 OR S29 OR S30 OR S31 OR S32 OR S33 OR S34 OR S35 OR S36 OR S37 OR S38 OR S39 OR S40 OR S41 OR S42 OR S43 OR S44 OR S45 OR S46 OR S47 OR S48 OR S49 OR S50 OR S51 OR S52 OR S53 OR S54 OR S55 OR S56

S58 “Community-Based Participatory Research”

S59 “Patient Participation”

S60 “Community Participation”

S61 “action research”

S62 “advisory”

S63 “co-creat*”

S64 “cocreat*”

S65 “co-design”

S66 “codesign”

S67 “co-produc*”

S68 “coproduc*”

S69 “co-evaluat*”

S70 “coevaluat*”

S71 “co-decision*”

S72 “codecision*”

S73 “co-method*”

S74 “engagement”

S75 “participatory design”

S76 “participatory research”

S77 “participatory health research”

S78 “patient participat*”

S79 “client participat*”

S80 (public and patient involvement)

S81 “user involvement”

S82 S58 OR S59 OR S60 OR S61 OR S62 OR S63 OR S64 OR S65 OR S66 OR S67 OR S68 OR S69 OR S70 OR S71 OR S72 OR S73 OR S74 OR S75 OR S76 OR S77 OR S78 OR S79 OR S80 OR S81

S83 MH “Aged+”

S84 MH “Aging+”

S85 “aged”

S86 “ageing”

S87 “aged individual*”

S88 “aged hospital patient*”

S89 “aged patient*”

S90 “aged client*”

S91 “aged worker*”

S92 “age 60”

S93 “age 65”

S94 “age 70”

S95 “age 75”

S96 “age 80”

S97 “aging adult*”

S98 “ageing adult*”

S99 “elder*”

S100 “frail elder*”

S101 “geriatric*”

S102 “late life”

S103 “master* athlete*”

S104 “old* adult*”

S105 “old* patient*”

S106 “old* client*”

S107 “old* people*”

S108 “old* veteran*”

S109 “senior*”

S110 “very elderl*”

S111 “60 and over”

S112 S83 OR S84 OR S85 OR S86 OR S87 OR S88 OR S89 OR S90 OR S91 OR S92 OR S93 OR S94 OR S95 OR S96 OR S97 OR S98 OR S99 OR S100 OR S101 OR S102 OR S103 OR S104 OR S105 OR S106 OR S107 OR S108 OR S109 OR S110 OR S111

S113 S57 AND S82 AND S112

**Embase (Ovid, 1974 to Present)**

Search conducted: February 28, 2022

Limits: No limits were applied to this search strategy

Number of studies retrieved: 10073

1 exp exercise/

2 exp Exercise Therapy/

3 exp Recreation/

4 exp Recreation Therapy/

5 exp Motor Activity/

6 exp Movement/

7 exp Sports/

8 exp Sports Equipment/

9 exp "Activities of Daily Living"/

10 exp Physical Fitness/

11 exp Walking/

12 exp Exercise Movement Techniques/

13 exp Physical Endurance/

14 abdominal exercis*.mp.

15 activit* of daily living.mp.

16 aerobic exercis*.mp.

17 anaerobic exercis*.mp.

18 arm exercis*.mp.

19 aquatic exercis*.mp.

20 breathing exercis*.mp.

21 cardiorespiratory fitness.mp.

22 closed kinetic chain exercis*.mp.

23 danc*.mp.

24 dynamic exercis*.mp.

25 exercis*.mp.

26 (exercise and fitness equipment).mp.

27 exercis* position.mp.

28 exercis* intensity.mp.

29 exercis* therapy.mp.

30 fitness.mp.

31 group exercis*.mp.

32 isokinetic exercis*.mp.

33 isotonic exercis*.mp.

34 isometric exercis*.mp.

35 kegel exercis*.mp.

36 leg exercis*.mp.

37 moderate to vigorous physical activ*.mp.

38 motor activit*.mp.

39 movement techniqu*.mp.

40 movement*.mp.

41 muscle exercis*.mp.

42 open kinetic chain exercis*.mp.

43 physical fit*.mp.

44 physical activ*.mp.

45 plyometric*.mp.

46 recreation*.mp.

47 resistance training.mp.

48 sport*.mp.

49 sport* equipment.mp.

50 static exercis*.mp.

51 stretching exercis*.mp.

52 swim*.mp.

53 treadmill exercis*.mp.

54 therapeutic exercis*.mp.

55 walk*.mp.

56 warm-up exercis*.mp.

57 1 or 2 or 3 or 4 or 5 or 6 or 7 or 8 or 9 or 10 or 11 or 12 or 13 or 14 or 15 or 16 or 17 or 18 or 19 or 20 or 21 or 22 or 23 or 24 or 25 or 26 or 27 or 28 or 29 or 30 or 31 or 32 or 33 or 34 or 35 or 36 or 37 or 38 or 39 or 40 or 41 or 42 or 43 or 44 or 45 or 46 or 47 or 48 or 49 or 50 or 51 or 52 or 53 or 54 or 55 or 56

58 exp Community-Based Participatory Research/

59 exp Patient Participation/

60 exp Community Participation/

61 action research.mp.

62 advisory.mp.

63 co-creat*.mp.

64 cocreat*.mp.

65 co-design.mp.

66 codesign.mp.

67 co-produc*.mp.

68 coproduc*.mp.

69 co-evaluat*.mp.

70 coevaluat*.mp.

71 co-decision*.mp.

72 codecision*.mp.

73 co-method*.mp.

74 community-based participatory research.mp.

75 community participation.mp.

76 engagement.mp.

77 participatory design.mp.

78 participatory research.mp.

79 participatory health research.mp.

80 patient participat*.mp.

81 client participat*.mp.

82 (public and patient involvement).mp.

83 user involvement.mp.

84 58 OR 59 OR 60 OR 61 OR 62 OR 63 OR 64 OR 65 OR 66 OR 67 OR 68 OR 69 OR 70 OR 71 OR 72 OR 73 OR 74 OR 75 OR 76 OR 77 OR 78 OR 79 OR 80 OR 81 OR 82 OR 83

85 exp Aged/

86 exp Aging/

87 aged.mp.

88 ageing.mp.

89 aged individual*.mp.

90 aged hospital patient*.mp.

91 aged patient*.mp.

92 aged client*.mp.

93 aged worker*.mp.

94 age 60.mp.

95 age 65.mp.

96 age 70.mp.

97 age 75.mp.

98 age 80.mp.

99 aging adult*.mp.

100 ageing adult*.mp.

101 elder*.mp.

102 frail elder*.mp.

103 geriatric*.mp.

104 late life.mp.

105 master* athlete*.mp.

106 old* adult*.mp.

107 old* patient*.mp.

108 old* client*.mp.

109 old* people*.mp.

110 old* veteran*.mp.

111 senior*.mp.

112 very elderl*.mp.

113 60 and over.mp.

114 85 OR 86 OR 87 OR 88 OR 89 OR 90 OR 91 OR 92 OR 93 OR 94 OR 95 OR 96 OR 97 OR 98 OR 99 OR 100 OR 101 OR 102 OR 103 OR 104 OR 105 OR 106 OR 107 OR 108 OR 109 OR 110 OR 111 OR 112 OR 113

115 57 AND 84 AND 114

**SPORTDiscus (EBSCOhost, 1830 to Present)**

Search conducted: February 28, 2022

Limits: No limits were applied to this search strategy

Number of studies retrieved: 782

S1 DE "EXERCISE" OR DE "ABDOMINAL exercises" OR DE "AEROBIC exercises" OR DE "ANAEROBIC exercises" OR DE "AQUATIC exercises" OR DE "ARM exercises" OR DE "BACK exercises" OR DE "BREATHING exercises" OR DE "BREEMA" OR DE "BUTTOCKS exercises" OR DE "CALISTHENICS" OR DE "CHAIR exercises" OR DE "CHEST exercises" OR DE "CIRCUIT training" OR DE "COMPOUND exercises" OR DE "COOLDOWN" OR DE "DO-in" OR DE "EXERCISE adherence" OR DE "EXERCISE for children" OR DE "EXERCISE for girls" OR DE "EXERCISE for men" OR DE "EXERCISE for middle-aged persons" OR DE "EXERCISE for older people" OR DE "EXERCISE for people with disabilities" OR DE "EXERCISE for women" OR DE "EXERCISE for youth" OR DE "EXERCISE therapy" OR DE "EXERCISE video games" OR DE "FACIAL exercises" OR DE "FALUN gong exercises" OR DE "FOOT exercises" OR DE "GYMNASTICS" OR DE "HAND exercises" OR DE "HATHA yoga" OR DE "HIP exercises" OR DE "ISOKINETIC exercise" OR DE "ISOLATION exercises" OR DE "ISOMETRIC exercise" OR DE "ISOTONIC exercise" OR DE "KNEE exercises" OR DE "LEG exercises" OR DE "LIANGONG" OR DE "METABOLIC equivalent" OR DE "MULAN quan" OR DE "MUSCLE strength" OR DE "PILATES method" OR DE "PLYOMETRICS" OR DE "QI gong" OR DE "REDUCING exercises" OR DE "RUNNING" OR DE "RUNNING -- Social aspects" OR DE "SCHOOL exercises & recreations" OR DE "SEXUAL exercises" OR DE "SHOULDER exercises" OR DE "STRENGTH training" OR DE "STRESS management exercises" OR DE "TAI chi" OR DE "TREADMILL exercise" OR DE "WHEELCHAIR workouts" OR DE "YOGA"

S2 DE "EXERCISE therapy" OR DE "EXERCISE therapy for children" OR DE "EXERCISE therapy for older people" OR DE "MENSENDIECK system" OR DE "ORTHOPTICS" OR DE "SWEDISH gymnastics" OR DE "THERAPEUTIC use of breathing exercises"

S3 DE "RECREATION" OR DE "ARCHITECTURE & recreation" OR DE "FAMILY recreation" OR DE "GAMES" OR DE "INDUSTRIAL recreation" OR DE "OUTDOOR recreation" OR DE "PLAY" OR DE "POPULAR culture" OR DE "RECREATION & state "OR DE "RECREATION for older people" OR DE "RECREATION programs" OR DE "RECREATIONAL sports" OR DE "RECREATIONAL therapy" OR DE "SCHOOL exercises & recreations" OR DE "SPORTS" OR DE "STUDENT recreation" OR DE "VOLUNTEER workers in recreation" OR DE "WILDLIFE-related recreation" OR DE "YOUTH recreation"

S4 DE “RECREATIONAL therapy” OR DE “PLAY therapy”

S5 “Motor Activity”

S6 “Movement”

S7 DE "SPORTS" OR DE "AERODYNAMICS in sports" OR DE "AERONAUTICAL sports" OR DE "AGE & sports" OR DE "AMATEUR sports" OR DE "ANIMAL sports" OR DE "ANTISEMITISM in sports" OR DE "AQUATIC sports" OR DE "BALL games" OR DE "BALLISTICS in sports" OR DE "BASEBALL" OR DE "BIOMECHANICS in sports" OR DE "COLLEGE sports" OR DE "COMBAT sports" OR DE "COMMUNICATION in sports" OR DE “CONTACT sports” OR DE “CROSS-training (Sports)” OR DE “DISC golf” OR DE “DISCRIMINATION in sports” OR DE “DOG sports” OR DE “DOPING in sports” OR DE “ENDURANCE sports” OR DE “EXTREME sports” OR DE “FANTASY sports” OR DE “FASCISM & sports” OR DE “FEMINISM & sports” OR DE “GAELIC games” OR DE “GAY Games” OR DE “GOODWILL Games” OR DE “GYMNASTICS” OR DE “HOCKEY” OR DE “HOMOPHOBIA in sports” OR DE “HYDRODYNAMIC in sports” OR DE “INDIVIDUAL sports” OR DE “KINEMATICS in sports” OR DE “KNIFE throwing” OR DE “LGBTQ+ people & sports” OR DE “LOG-chopping (Sports)” OR DE “MASCULINITY in sports” OR DE “MASS media & sports” OR DE “MILITARY sports” OR DE “MINORITIES in sports” OR DE “MOTION pictures in sports” OR DE “MOTORSPORTS” OR DE “NATIONAL socialism & sports” OR DE “NATIONALISM & sports” OR DE “NONVERBAL communication in sports” OR DE “OLYMPIC Games” OR DE “PARKOUR” OR DE “PHYSICS in sports” OR DE “PRESIDENTS – Sports” OR DE “PROFESSIONAL sports” OR DE “ PROFESSIONALISM in sports” OR DE “RACISM in sports” OR DE “RACKET games” OR DE “RADAR in sports” OR DE “RECREATIONAL sports” OR DE “REGIONALISM & sports” OR DE “ROBOTICS in sports” OR DE “RODEOS” OR DE “ROLLER skating” OR DE “SCHOOL sports” OR DE “SENIOR Olympics” OR DE “SEXUAL harassment in sports” OR DE “SHOOTING (Sports)” OR DE “SHUTOUTS (Sports)” OR DE “SKATEBOARDING” OR DE “SOCIALISM & sports” OR DE “SOFTBALL” OR DE “SPORT for all” OR DE “ SPORTS & state” OR DE “SPORTS & technology” OR DE “SPORTS & theater” OR DE “SPORTS & tourism” OR DE “SPORTS for children” OR DE “SPORTS for girls” OR DE “SPORTS for older people” OR DE “SPORTS for people with disabilities” OR DE “SPORTS for youth” OR DE “SPORTS forecasting” OR DE “SPORTS in antiquity” OR DE “SPORTS penalties” OR DE “SPORTS photography” OR DE “SPORTS rivalries” OR DE “SPORTS teams” OR DE “SPORTS tourism” OR DE “STEREOTYPES in sports” OR DE “TARGETS (Sports)” OR DE “TEAM sports” OR DE “TEAMWORK (Sports)” OR DE “TELEVISION & sports” OR DE “TRACEURS” OR DE “VIDEO tapes in sports” OR DE “VIOLENCE in sports” OR DE “WINTER sports” OR DE “WOMEN’S sports”

S8 DE “SPORTING goods industry” OR DE “BASEBALL equipment industry” OR DE “BICYCLE industry” OR DE “BODYBUILDING industry” OR DE “CAMPING equipment industry” OR DE “CRICKET equipment industry” OR DE “EXERCISE equipment industry” OR DE “FOOTBALL equipment industry” OR DE “GOLF equipment industry” OR DE “HOCKEY equipment industry” OR DE “RACQUETBALL industry” OR DE “SKIING equipment industry” OR DE “SOCCER equipment industry” OR DE “SOFTBALL equipment industry” OR DE “SPORTSWEAR industry” OR DE “SURFBOARD industry” OR DE “TENNIS industry”

S9 DE “ACTIVITIES of daily living”

S10 DE "PHYSICAL fitness" OR DE "ANAEROBIC exercises" OR DE "ASTROLOGY & physical fitness" OR DE "BODYBUILDING" OR DE "CARDIOPULMONARY fitness" OR DE "CARDIOVASCULAR fitness" OR DE "CIRCUIT training" OR DE "COMPOUND exercises" OR DE "EXERCISE tolerance" OR DE "ISOLATION exercises" OR DE "LIANGONG" OR DE "MUSCLE strength" OR DE "PERIODIZATION training" OR DE "PHYSICAL fitness for children" OR DE "PHYSICAL fitness for girls" OR DE "PHYSICAL fitness for men" OR DE "PHYSICAL fitness for older people" OR DE "PHYSICAL fitness for people with disabilities" OR DE "PHYSICAL fitness for women" OR DE "PHYSICAL fitness for youth" OR DE "SPORT for all"

S11 DE “WALKING” OR DE “FITNESS walking” OR DE “GAIT in humans” OR DE “HIKING” OR DE “LONG distance walking” OR DE “VIERDAAGSE (Walking event)”

S12 “Exercise Movement Techniques”

S13 “Physical Endurance”

S14 “abdominal exercis*”

S15 “activit* of daily living”

S16 “aerobic exercis*”

S17 “anaerobic exercis*”

S18 “arm exercis*”

S19 “aquatic exercis*”

S20 “breathing exercis*”

S21 “cardiorespiratory fitness”

S22 “closed kinetic chain exercis*”

S23 “danc*”

S24 “dynamic exercis*”

S25 “exercis*”

S26 (exercise and fitness equipment)

S27 “exercis* position”

S28 “exercis* intensity”

S29 “exercis* therapy”

S30 “fitness”

S31 “group exercis*”

S32 “isokinetic exercis*”

S33 “isotonic exercis*”

S34 “isometric exercis*”

S35 “kegal exercis*”

S36 “leg exercis*”

S37 “moderate to vigorous physical activ*”

S38 “motor activit*”

S39 “movement techniqu*”

S40 “movement*”

S41 “muscle exercis*”

S42 “open kinetic chain exercis*”

S43 “physical fit*”

S44 “physical activ*”

S45 “plyometric*”

S46 “recreation*”

S47 “sport*”

S48 “sport* equipment”

S49 “static exercis*”

S50 “stretching exercis*”

S51 “swim*”

S52 “treadmill exercis*”

S53 “therapeutic exercis*”

S54 “walk*”

S55 “warm-up exercis*”

S56 “resistance training”

S57 S1 OR S2 OR S3 OR S4 OR S5 OR S6 OR S7 OR S8 OR S9 OR S10 OR S11 OR S12 OR S13 OR S14 OR S15 OR S16 OR S17 OR S18 OR S19 OR S20 OR S21 OR S22 OR S23 OR S24 OR S25 OR S26 OR S27 OR S28 OR S29 OR S30 OR S31 OR S32 OR S33 OR S34 OR S35 OR S36 OR S37 OR S38 OR S39 OR S40 OR S41 OR S42 OR S43 OR S44 OR S45 OR S46 OR S47 OR S48 OR S49 OR S50 OR S51 OR S52 OR S53 OR S54 OR S55 OR S56

S58 “Patient Participation”

S59 DE “RECREATION & state” OR DE “COMMUNITY recreation programs (Government)”

S60 “action research”

S61 “advisory”

S62 “co-creat*”

S63 “cocreat*”

S64 “co-design”

S65 “codesign”

S66 “co-produc*”

S67 “coproduc*”

S68 “co-evaluat*”

S69 “coevaluat*”

S70 “co-decision*”

S71 “codecision”

S72 “co-method*”

S73 “community-based participatory research”

S74 “community participation”

S75 “engagement”

S76 “participatory design”

S77 “participatory research”

S78 “participatory health research”

S79 “patient participat*”

S80 “client participat*”

S81 (public and patient involvement)

S82 “user involvement”

S83 S58 OR S59 OR S60 OR S61 OR S62 OR S63 OR S64 OR S65 OR S66 OR S67 OR S68 OR S69 OR S70 OR S71 OR S72 OR S73 OR S74 OR S75 OR S76 OR S77 OR S78 OR S79 OR S80 OR S81 OR S82

S84 DE “OLDER people” OR DE “EXERCISE for older people” OR DE “PHYSICAL education for older people” OR DE “PHYSICAL fitness for older people” OR DE “SPORTS for older people”

S85 DE “AGING” OR DE “IMMUNE system aging” OR DE “MUSCLE aging”

S86 “aged”

S87 “ageing”

S88 “aged individual*”

S89 “aged hospital patient*”

S90 “aged patient*”

S91 “aged client*”

S92 “aged worker*”

S93 “age 60”

S94 “age 65”

S95 “age 70”

S96 “age 75”

S97 “age 80”

S98 “aging adult*”

S99 “ageing adult*”

S100 “elder*”

S101 “frail elder*”

S102 “geriatric*”

S103 “late life”

S104 “master* athlete*”

S105 “old* adult*”

S106 “old* patient*”

S107 “old* client*”

S108 “old* people*”

S109 “old* veteran*”

S110 “senior*”

S111 “very elderl*”

S112 “60 and over”

S113 S84 OR S85 OR S86 OR S87 OR S88 OR S89 OR S90 OR S91 OR S92 OR S93 OR S94 OR S95 OR S96 OR S97 OR S98 OR S99 OR S100 OR S101 OR S102 OR S103 OR S104 OR S105 OR S106 OR S107 OR S108 OR S109 OR S110 OR S111 OR S112

S114 S57 AND S83 AND S113

S115 S57 AND S83 AND S113
