# Supplementary material for: The effects of co-designed physical activity interventions in older adults: A systematic review and meta-analysis
Source: PLoS One. 2024 May 10;19(5):e0297675. doi: 10.1371/journal.pone.0297675 (PMC11086838; doi:10.1371/journal.pone.0297675)
Supplement: S2 File — (DOCX) [file pone.0297675.s002.docx]

S2 File. PRISMA checklist.

| **Section/topic** | **#** | **Checklist item** | **Reported on page #** |
| --- | --- | --- | --- |
| **TITLE** | | |  |
| Title | 1 | Identify the report as a systematic review, meta-analysis, or both.  Title: “Measuring the global burden of chikungunya and Zika viruses: a systematic review” | Page 1 |
| **ABSTRACT** | | |  |
| Structured summary | 2 | Provide a structured summary including, as applicable: background; objectives; data sources; study eligibility criteria, participants, and interventions; study appraisal and synthesis methods; results; limitations; conclusions and implications of key findings; systematic review registration number.  These requirements are met throughout the text of the Abstract | Page 2 |
| **INTRODUCTION** | | |  |
| Rationale | 3 | Describe the rationale for the review in the context of what is already known.  The most recent Global Burden of Disease (GBD) estimates published by the World Health Organization (WHO) include assessments of only two arboviral diseases: dengue and yellow fever [33]. For other arboviruses, including CHIKV and ZIKV, all cause-specific …represent substantial health deficits omitted in international disease burden reports, and are consequently not included in top-level discussions of disease control priorities [34]. With evidence from the past decade indicating the high incidence and frequency of disabling sequelae due to CHIKV and ZIKV, it is important to address this gap in knowledge and to quantify the impacts of the two viruses | Page 3 |
| Objectives | 4 | Provide an explicit statement of questions being addressed with reference to participants, interventions, comparisons, outcomes, and study design (PICOS).  In the present study, we reviewed the available data detailing CHIKV and ZIKV infections and their impact in order to estimate the disability-adjusted life years (DALYs) lost globally as a result of their spread over the last decade. | Page 3-4 |
| **METHODS** | | |  |
| Protocol and registration | 5 | Indicate if a review protocol exists, if and where it can be accessed (e.g., Web address), and, if available, provide registration information including registration number. | Page 3  CRD42022314217 |
| Eligibility criteria | 6 | Specify study characteristics (e.g., PICOS, length of follow-up) and report characteristics (e.g., years considered, language, publication status) used as criteria for eligibility, giving rationale.  We systematically reviewed the available published literature and official reports on CHIKV and ZIKV. Publications were required to meet three inclusion criteria: 1) discussion of complications that lead to mortality or prolonged morbidity; 2) focus on population-based information; and 3) reported data collected between 2010 and 2019. In order to assess the population-level impacts of endemic disease, studies involving travelers from non-endemic areas and all case reports were excluded from analysis… Bibliographies of selected publications were also searched for additional reports. English, Spanish, French, and Portuguese reports were screened. | Page 4 |
| Information sources | 7 | Describe all information sources (e.g., databases with dates of coverage, contact with study authors to identify additional studies) in the search and date last searched.  Searching was initiated with the use of the arbovirus name and the terms “outbreak(s),” “complication(s),” “disability,” “quality of life,” “morbidity,” “mortality,” “DALY,” and “QALY” in PubMed, Google Scholar, LILACS, African Journals Online, SCIELO, and Web of Science. | Page 4 |
| Search | 8 | Present full electronic search strategy for at least one database, including any limits used, such that it could be repeated.  Searching was initiated with the use of the arbovirus name and the terms “outbreak(s),” “complication(s),” “disability,” “quality of life,” “morbidity,” “mortality,” “DALY,” and “QALY” in PubMed, Google Scholar, LILACS, African Journals Online, SCIELO, and Web of Science. | S1 File |
| Study selection | 9 | State the process for selecting studies (i.e., screening, eligibility, included in systematic review, and, if applicable, included in the meta-analysis).  Publications were required to meet three inclusion criteria: 1) discussion of complications that lead to mortality or prolonged morbidity; 2) focus on population-based information; and 3) reported data collected between 2010 and 2019. In order to assess the population-level impacts of endemic disease, studies involving travelers from non-endemic areas and all case reports were excluded from analysis | Page 4,  Fig 1 |
| Data collection process | 10 | Describe method of data extraction from reports (e.g., piloted forms, independently, in duplicate) and any processes for obtaining and confirming data from investigators.  In order to derive DALY estimates for CHIKV and ZIKV, we extracted from the included reports estimates of incidence, mortality, average age at death, and, for nonlethal cases, information on the duration and severity of acute and chronic symptoms. Study populations and dates were carefully reviewed to ensure that any duplicate data were not included more than once. | Page 5 |
| Data items | 11 | List and define all variables for which data were sought (e.g., PICOS, funding sources) and any assumptions and simplifications made.  In order to derive DALY estimates for CHIKV and ZIKV, we extracted from the included reports estimates of incidence, mortality, average age at death, and, for nonlethal cases, information on the duration and severity of acute and chronic symptoms. Study populations and dates were carefully reviewed to ensure that any duplicate data were not included more than once. | Page 4, 5 |
| Risk of bias in individual studies | 12 | Describe methods used for assessing risk of bias of individual studies (including specification of whether this was done at the study or outcome level), and how this information is to be used in any data synthesis. | Page 5 |
| Summary measures | 13 | State the principal summary measures (e.g., risk ratio, difference in means).  We report our DALY estimates in two formats: discrete approximations calculated using inputs derived from weighted averaging of the variables extracted from included publications, and ranges based on the variability of credible input values contained in those reports. | Page 5 |
| Synthesis of results | 14 | Describe the methods of handling data and combining results of studies, if done, including measures of consistency (e.g., I^2^) for each meta-analysis. | Page 5 |
| Risk of bias across studies | 15 | Specify any assessment of risk of bias that may affect the cumulative evidence (e.g., publication bias, selective reporting within studies).  These infections, combined with those documented as “suspected” or “likely” by the respective reporting bodies, served as the upper bound. In light of the recognized trends of under-reporting [38,39], these larger case count values were used as the inputs for our calculations of discrete estimates.  The wide ranges of our estimated burden values, presented in Tables 4 and 5, reflect the current uncertainty in predicting the short- and long-term outcomes of infections, inconsistencies in published findings, and inherent challenges in generating yearly DALY estimates for arboviral diseases… Due to inconsistencies in diagnostics and likely under-reporting in endemic areas, it is impossible at this time to know precisely the number of symptomatic infections globally or their associated cause-specific deaths. | Page 5 |
| Additional analyses | 16 | Describe methods of additional analyses (e.g., sensitivity or subgroup analyses, meta-regression), if done, indicating which were pre-specified. | N/A |
| **RESULTS** | | |  |
| Study selection | 17 | Give numbers of studies screened, assessed for eligibility, and included in the review, with reasons for exclusions at each stage, ideally with a flow diagram.  Of 7,877 studies identified, 916 were screened and 21 were selected for inclusion (Fig 1). | Page 4, Fig 1 |
| Study characteristics | 18 | For each study, present characteristics for which data were extracted (e.g., study size, PICOS, follow-up period) and provide the citations.  Excel file contains all included studies, populations, sample sizes, and citations | Page 6-7, S3 Table |
| Risk of bias within studies | 19 | Present data on risk of bias of each study and, if available, any outcome level assessment (see item 12). | Page 11, S4 Figure |
| Results of individual studies | 20 | For all outcomes considered (benefits or harms), present, for each study: (a) simple summary data for each intervention group (b) effect estimates and confidence intervals, ideally with a forest plot. | Page 11-13 |
| Synthesis of results | 21 | Present results of each meta-analysis done, including confidence intervals and measures of consistency. | Page 10-14, Fig 2, 3 |
| Risk of bias across studies | 22 | Present results of any assessment of risk of bias across studies (see Item 15). | Page 11, S4 Figure |
| Additional analysis | 23 | Give results of additional analyses, if done (e.g., sensitivity or subgroup analyses, meta-regression [see Item 16]). | N/A |
| **DISCUSSION** | | |  |
| Summary of evidence | 24 | Summarize the main findings including the strength of evidence for each main outcome; consider their relevance to key groups (e.g., healthcare providers, users, and policy makers). In our current analysis we estimated that the virus has caused the annualized loss of over 158,000 DALYs in AMRO since its emergence. This value confirms that CHIKV is among the most problematic arboviruses in the region, causing a burden second only to that of DENV, which the WHO estimated to cause the loss of 203,000 DALYs in its 2016 GBD report [36]. We estimate that ZIKV has caused an annualized DALY burden of 85,000 in AMRO since its emergence there in 2015. This estimate aligns with those previously published for Latin America, and far exceeds the 23,000 DALYs associated with yellow fever in AMRO [36,69]… The burden caused by these viruses should be included in routine reports and regularly acknowledged in discussions of policy and containment priorities. | Page 14-16 |
| Limitations | 25 | Discuss limitations at study and outcome level (e.g., risk of bias), and at review-level (e.g., incomplete retrieval of identified research, reporting bias).  The wide ranges of our estimated burden values, presented in Tables 4 and 5, reflect the current uncertainty in predicting the short- and long-term outcomes of infections, inconsistencies in published findings, and inherent challenges in generating yearly DALY estimates for arboviral diseases… Due to inconsistencies in diagnostics and likely under-reporting in endemic areas, it is impossible at this time to know precisely the number of symptomatic infections globally or their associated cause-specific deaths. Likewise, due to limited long-term data, it is currently unclear how many chronic cases either virus has caused, or the duration for which patients generally experience long-term complications. In particular, more follow-up will be needed to ascertain the lifetime prognosis of CZS, a condition which has only been studied since its discovery in 2015 [21,24]. | Page 16 |
| Conclusions | 26 | Provide a general interpretation of the results in the context of other evidence, and implications for future research.  Our DALY estimates for CHIKV align with those reported by Cardona-Espina et al., who analyzed chronic CHIKV-linked burden in Latin America in 2014 and noted that DALYs attributable to CHIKV likely outweighed those of any other arbovirus in the region that year [68]. In our current analysis we estimated that the virus has caused the annualized loss of over 158,000 DALYs in AMRO since its emergence. This value confirms that CHIKV is among the most problematic arboviruses in the region, causing a burden second only to that of DENV, which the WHO estimated to cause the loss of 203,000 DALYs in its 2016 GBD report [36]. | Page 17 |
| **FUNDING** | | |  |
| Funding | 27 | Describe sources of funding for the systematic review and other support (e.g., supply of data); role of funders for the systematic review.  CJP is supported by the Stanford Medical Scholars Research Program. ADL is supported by the National Institutes of Health through grant #R01 AI102918 and a philanthropic gift to Stanford University in support of research on the neurodevelopmental impacts of Zika virus in Grenada. The funders did not play any role in the study design, data collection, analysis, or interpretation of the results, nor did they participate in the preparation of this manuscript work or the decision to publish this work. | Page 17 |

*From:*  Moher D, Liberati A, Tetzlaff J, Altman DG, The PRISMA Group (2009). Preferred Reporting Items for Systematic Reviews and Meta-Analyses: The PRISMA Statement. PLoS Med 6(7): e1000097. doi:10.1371/journal.pmed1000097

For more information, visit: **www.prisma-statement.org**.
