# Supplementary material for: The effects of co-designed physical activity interventions in older adults: A systematic review and meta-analysis
Source: PLoS One. 2024 May 10;19(5):e0297675. doi: 10.1371/journal.pone.0297675 (PMC11086838; doi:10.1371/journal.pone.0297675)
Supplement: S1 Table — (DOCX) [file pone.0297675.s003.docx]

S1 Table. List of excluded studies and reasons for exclusion.

| **Reference** | **Reason for Exclusion** |
| --- | --- |
| C.M. T, G.M. W, J.L. W, T.M. A, F.F. D, Tucker CM, et al. A CBPR Study to Test the Impact of a Church-Based Health Empowerment Program on Health Behaviors and Health Outcomes of Black Adult Churchgoers. Journal of racial and ethnic health disparities [Internet]. 2017;4(1):70–8. | Not older adult |
| Collins ES, Buchholz SW, Cranford J, McCrory MA. A Church-based Culturally Sensitive Physical Activity Intervention in African American Women. Western Journal of Nursing Research [Internet]. 2021;43(6):563–71. | Not older adult |
| AH R. A comparison of physician based exercise counseling protocols: a pilot study. Does physical activity counseling provided by a physician in the form of verbal, written or combination delivery increase physical activity in a sedentary geriatric pop... [Internet]. Comparison of Physician Based Exercise Counseling Protocols: A Pilot Study. Does Physical Activity Counseling Provided by a Physician in the Form of Verbal, Written or Combination Delivery Increase Physical Activity in a Sedentary Geriatric Population... University of South Carolina; 2007. p. 134 p. | Not older adult |
| Campbell KJ, Louie PK, Bohl DD, Edmiston T, Mikhail C, Li J, et al. A Novel, Automated Text-Messaging System Is Effective in Patients Undergoing Total Joint Arthroplasty. The Journal of bone and joint surgery American volume [Internet]. 2019;101(2):145–51. | Not older adult |
| Vetrovsky T, Cupka J, Dudek M, Kuthanova B, Vetrovska K, Capek V, et al. A pedometer-based walking intervention with and without email counseling in general practice: a pilot randomized controlled trial. BMC public health [Internet]. 2018;18(1):635–635. | Not older adult |
| Islam NS, Zanowiak JM, Wyatt LC, Chun K, Lee L, Kwon SC, et al. A randomized-controlled, pilot intervention on diabetes prevention and healthy lifestyles in the New York City Korean community. Journal of community health [Internet]. 2013;38(6):1030–41. | Not older adult |
| A.C. B, Betts AC, Froehlich-Grobe K. Accessible weight loss: Adapting a lifestyle intervention for adults with impaired mobility. Disability and health journal [Internet]. 2017;10(1):139–44. | Not older adult |
| Murphy SM, Edwards RT, Williams N, Raisanen L, Moore G, Linck P, et al. An evaluation of the effectiveness and cost effectiveness of the National Exercise Referral Scheme in Wales, UK: a randomised controlled trial of a public health policy initiative. Journal of epidemiology and community health [Internet]. 2012;66(8):745–53. | Not older adult |
| Armstrong KA, Metlay JP. Annals Clinical Decision Making: Communicating Risk and Engaging Patients in Shared Decision Making. Annals of internal medicine [Internet]. 2020;172(10):688–92. | Not older adult |
| Johnson R, Robertson W, Towey M, Stewart-Brown S, Clarke A. Changes over time in mental well-being, fruit and vegetable consumption and physical activity in a community-based lifestyle intervention: a before and after study. Public health [Internet]. 2017;146:118–25. | Not older adult |
| Estabrooks PA, Bradshaw M, Dzewaltowski DA, Smith-Ray RL. Determining the impact of Walk Kansas: applying a team-building approach to community physical activity promotion. Annals of behavioral medicine : a publication of the Society of Behavioral Medicine [Internet]. 2008;36(1):1–12. | Not older adult |
| K.D. H, A. W, J. S, P. B, H.-H. H, J. B, et al. Developing a Community-Based Tailored Exercise Program for People With Severe and Persistent Mental Illness. Progress in community health partnerships: research, education, and action [Internet]. 2015;9(2):213–27. | Not older adult |
| V.E. S, S. H, N.E. W, J.R. K, M. M, M. U, et al. Developing a group intervention to manage fatigue in rheumatoid arthritis through modifying physical activity. BMC Musculoskeletal Disorders [Internet]. 2019;20(1):194–194. | Not older adult |
| Graham CR, Larstone R, Griffiths B, de Leeuw S, Anderson L, Powell-Hellyer S, et al. Development and Evaluation of Innovative Peer-Led Physical Activity Programs for Mental Health Service Users. The Journal of nervous and mental disease [Internet]. 2017;205(11):840–7. | Not older adult |
| A.J. S, B.A. I, G.B. M, C. B, D. C, R. D, et al. Effectiveness of a walking group intervention to promote physical activity and cardiovascular health in predominantly non-Hispanic black and Hispanic urban neighborhoods: findings from the walk your heart to health intervention. Health education & behavior : the official publication of the Society for Public Health Education [Internet]. 2015;42(3):380–92. | Not older adult |
| Li Z, Chen Q, Yan J, Liang W, Wong WCW, Z. L, et al. Effectiveness of motivational interviewing on improving Care for Patients with type 2 diabetes in China: A randomized controlled trial. BMC health services research [Internet]. 2020;20(1):57–57. | Not older adult |
| Luhr K, Eldh AC, Theander K, Holmefur M. Effects of a self-management programme on patient participation in patients with chronic heart failure or chronic obstructive pulmonary disease: A randomized controlled trial. European journal of cardiovascular nursing : journal of the Working Group on Cardiovascular Nursing of the European Society of Cardiology [Internet]. 2019;18(3):185–93. | Not older adult |
| A. S, E. R, J. Z, B. T, A. P. Engagement in e-cycling and the self- management of type 2 diabetes: A qualitative study in primary care. BJGP Open [Internet]. 2019;3(2). | Not older adult |
| Quinn E, O’Hara BJ, Ahmed N, Winch S, McGill B, Banovic D, et al. Enhancing the get healthy information and coaching service for Aboriginal adults: Evaluation of the process and impact of the program. International journal for equity in health [Internet]. 2017;16(1):168–168. | Not older adult |
| Bazzano AT, Zeldin AS, Diab IRS, Garro NM, Allevato NA, Lehrer D, et al. The Healthy Lifestyle Change Program: a pilot of a community-based health promotion intervention for adults with developmental disabilities. American Journal of Preventive Medicine [Internet]. 2009;37(6 Suppl 1):S201-8. | Not older adult |
| Woods G, Levinson AH, Jones G, Kennedy RL, Johnson LC, Tran ZV, et al. The Living Well by Faith Health and wellness program for African Americans: an exemplar of community-based participatory research. Ethnicity & disease [Internet]. 2013;23(2):223–9. | Not older adult |
| Alley SJ, Kolt GS, Duncan MJ, Caperchione CM, Savage TN, Maeder AJ, et al. International Journal of Behavioral Nutrition and Physical Activity The effectiveness of a web 2.0 physical activity intervention in older adults - a randomised controlled trial. International Journal of Behavioral Nutrition & Physical Activity [Internet]. 2018;15(1):1-N.PAG. | Not older adult |
| Richert J, Lippke S, Ziegelmann JP, J. R, S. L. Intervention-engagement and its role in the effectiveness of stage-matched interventions promoting physical exercise. Research in sports medicine (Print) [Internet]. 2011;19(3):145–61. | Not older adult |
| Ries AV, Blackman LT, Page RA, Gizlice Z, Benedict S, Barnes K, et al. Goal setting for health behavior change: evidence from an obesity intervention for rural low-income women. Rural and remote health [Internet]. 2014;14:2682–2682. | Not older adult |
| Krieger J, Rabkin J, Sharify D, Song L, J. K, J. R, et al. High point walking for health: creating built and social environments that support walking in a public housing community. American journal of public health [Internet]. 2009;99 Suppl 3:S593-599. | Not older adult |
| K. T, J. V. Increasing physical activity efficiently: An experimental pilot study of a website and mobile phone intervention. International Journal of Telemedicine and Applications [Internet]. 2014;746232–746232. | Not older adult |
| Sherwood NE, Crain AL, Martinson BC, Hayes MG, Anderson JD, Clausen JM, et al. Keep it off: a phone-based intervention for long-term weight-loss maintenance. Contemporary clinical trials [Internet]. 2011;32(4):551–60. | Not older adult |
| II WL, VR O. Mobilizing and maintaining a coalition to promote physical activity among African Americans in Southeast Stockton, California. Journal of Health Education [Internet]. 1999;30(2):S31-6. | Not older adult |
| Driver S, Reynolds M, Kramer K. Modifying an evidence-based lifestyle programme for individuals with traumatic brain injury. Brain injury [Internet]. 2017;31(12):1612–6. | Not older adult |
| Granner ML, Sharpe PA, Burroughs EL, Fields R, Hallenbeck J. Newspaper content analysis in evaluation of a community-based participatory project to increase physical activity. Health education research [Internet]. 2010;25(4):656–67. | Not older adult |
| Morris ME, Slade SC, Wittwer JE, Blackberry I, Haines S, Hackney ME, et al. Online Dance Therapy for People With Parkinson’s Disease: Feasibility and Impact on Consumer Engagement. Neurorehabilitation and neural repair [Internet]. 2021;35(12):1076–87. | Not older adult |
| Cohen DA, Han B, Derose KP, Williamson S, Marsh T, McKenzie TL. Physical activity in parks: A randomized controlled trial using community engagement. American journal of preventive medicine [Internet]. 2013;45(5):590–7. | Not older adult |
| Buckley BJR, Thijssen DHJ, Murphy RC, Graves LEF, Whyte G, Gillison F, et al. Preliminary effects and acceptability of a co-produced physical activity referral intervention. Health Education Journal [Internet]. 2019;78(8):869–84. | Not older adult |
| Andreae SJ, Halanych JH, Cherrington A, Safford MM. Recruitment of a rural, southern, predominantly African-American population into a diabetes self management trial. Contemporary clinical trials [Internet]. 2012;33(3):499–506. | Not older adult |
| Pullen T, Bottorff JL, Sabiston CM, Campbell KL, Eves ND, Ellard SL, et al. Utilizing RE-AIM to examine the translational potential of Project MOVE, a novel intervention for increasing physical activity levels in breast cancer survivors. Translational behavioral medicine [Internet]. 2019;9(4):646–55. | Not older adult |
| Beekman E, Braun SM, Ummels D, van Vijven K, Moser A, Beurskens AJ. Validity, reliability and feasibility of commercially available activity trackers in physical therapy for people with a chronic disease: a study protocol of a mixed methods research. Pilot and feasibility studies [Internet]. 2017;3:64–64. | Not older adult |
| Handley M, MacGregor K, Schillinger D, Sharifi C, Wong S, Bodenheimer T. Using action plans to help primary care patients adopt healthy behaviors: a descriptive study. Journal of the American Board of Family Medicine : JABFM [Internet]. 2006;19(3):224–31. | Not older adult |
| Elsworth C, Winward C, Sackley C, Meek C, Freebody J, Esser P, et al. Supported community exercise in people with long-term neurological conditions: a phase II randomized controlled trial. Clinical Rehabilitation [Internet]. 2011;25(7):588–98. | Not older adult |
| Giachello AL, Arrom JO, Davis M, et al. Reducing diabetes health disparities through community-based participatory action research: the Chicago Southeast Diabetes Community Action Coalition. *Public Health Rep*. 2003;118(4):309-323. doi:10.1093/phr/118.4.309 | Not older adult |
| A. T, L. H, Tiedemann A, Hassett L, Sherrington C. A novel approach to the issue of physical inactivity in older age. Preventive medicine reports [Internet]. 2015;2:595–7. | No co-design |
| B.C. B, J.M. H, B.T. B, A. P, A.M. R, B.A. T, et al. A Patient-Centered Activity Regimen Improves Participation in Physical Activity Interventions in Advanced Stage Lung Cancer. Integrative Cancer Therapies [Internet]. 2018;17(3):921–7. | No co-design |
| Matz-Costa C, Lubben J, Lachman ME, Lee H, Choi YJ, C. MC, et al. A Pilot Randomized Trial of an Intervention to Enhance the Health-Promoting Effects of Older Adults’ Activity Portfolios: The Engaged4Life Program. Journal of gerontological social work [Internet]. 2018;61(8):792–816. | No co-design |
| Lyons K, Zajack A, Greer M, Chaimoy H, Dieckmann N, Carter J. 174 Benefits of a Self-Management Program for the Older Couple Living with Parkinson’s Disease: A Pilot Study. Age and Ageing [Internet]. 2019;48(Supplement):iii17–65. | No co-design |
| Chew-Graham CA, Lovell K, Roberts C, Baldwin R, Morley M, Burns A, et al. A randomised controlled trial to test the feasibility of a collaborative care model for the management of depression in older people. The British journal of general practice : the journal of the Royal College of General Practitioners [Internet]. 2007;57(538):364–70. | No co-design |
| Yamamoto S, Ishii D, Noguchi A, Tanamachi K, Okamoto Y, Takasaki Y, et al. A Short-Duration Combined Exercise and Education Program to Improve Physical Function and Social Engagement in Community-Dwelling Elderly Adults. International quarterly of community health education [Internet]. 2020;40(4):281–7. | No co-design |
| A.L. H, S. G, B.M. L. A telephone-delivered lifestyle intervention for colorectal cancer survivors “CanChange”: A pilot study. Psycho-Oncology [Internet]. 2009;18(4):449–55. | No co-design |
| A. DA, K. Z, S. N, A.-S. F, Niklasson J.  AO  - D. Almevall A, ORCID: http://orcid.org/0000-0002-1022-8741. Accepting the inevitable: A mixed method approach with assessment and perceptions of well-being in very old persons within the northern Sweden Silver-MONICA study. Archives of Gerontology and Geriatrics [Internet]. 2021;92:104275–104275. | No co-design |
| K.E. F, S. K, D. Z, M. H. Adherence to yoga and exercise interventions in a 6-month clinical trial. BMC Complementary and Alternative Medicine [Internet]. 2007;7:37–37. | No co-design |
| S. B, T. H, J. K, I. M, U.A. M, T. L, et al. An evidence-based shared decision making programme on the prevention of myocardial infarction in type 2 diabetes: protocol of a randomised-controlled trial. BMC family practice [Internet]. 2013;14:155–155. | No co-design |
| A. S, P. K, C. D, R. S, J.C. L. Association of motivations and barriers with participation and performance in a pedometer-based intervention. Nephrology Dialysis Transplantation [Internet]. 2020;35(8):1405–11. | No co-design |
| A. N, S. M, C.G. T, D.G. DR, C. G, B. M, et al. B-PO01-095 IMPACT OF DIGITAL MONITORING ON COMPLIANCE AND OUTCOME OF LIFESTYLE CHANGE MEASURES IN PATIENTS WITH COEXISTENT ATRIAL FIBRILLATION AND OBESITY. Heart Rhythm [Internet]. 2021;18(8 Supplement):S88–9. | No co-design |
| ARNAUTOVSKA U, O’CALLAGHAN F, HAMILTON K. Behaviour change techniques to facilitate physical activity in older adults: what and how. Ageing & Society [Internet]. 2018;38(12):2590–616. | No co-design |
| Liu-Ambrose TYL, Khan KM, Eng JJ, Gillies GL, Lord SR, McKay HA. Beneficial effects of group-based exercises on fall risk profile and physical activity persist 1 year postintervention in older women with low bone mass: follow-up after withdrawal of exercise. Journal of the American Geriatrics Society [Internet]. 2005;53(10):1767–73. | No co-design |
| C. MM, A.E. H, C.F. M, A. M, J.A. A, C.J. H, et al. Community Participation by People with Chronic Obstructive Pulmonary Disease. COPD: Journal of Chronic Obstructive Pulmonary Disease [Internet]. 2021;18(5):533–40. | No co-design |
| Han BH, Sadarangani T, Wyatt LC, Zanowiak JM, Kwon SC, Trinh-Shevrin C, et al. Correlates of Physical Activity Among Middle-Aged and Older Korean Americans at Risk for Diabetes. Journal of nursing scholarship : an official publication of Sigma Theta Tau International Honor Society of Nursing [Internet]. 2016;48(1):48–57. | No co-design |
| Frigault JS, Giles AR, Walker J. Culturally Safe Falls Prevention Program for Inuvialuit Elders in Inuvik, Northwest Territories, Canada: Considerations for Development and Implementation. Canadian Journal on Aging [Internet]. 2020;39(2):190–205. | No co-design |
| Mikkelsen MK, Lund CM, Vinther A, Tolver A, Johansen JS, Chen I, et al. Effects of a 12-Week Multimodal Exercise Intervention Among Older Patients with Advanced Cancer: Results from a Randomized Controlled Trial. Oncologist [Internet]. 2021; | No co-design |
| A.P. G, S. T, S. H, C. S, C. C. Effects of a home-based telephone-supported physical activity program on physical function among older adults with chronic low back pain. Arthritis and Rheumatology [Internet]. 2017;69(Supplement 10). | No co-design |
| Graumlich JF, Wang H, Madison A, Wolf MS, Kaiser D, Dahal K, et al. Effects of a Patient-Provider, Collaborative, Medication-Planning Tool: A Randomized, Controlled Trial. Journal of diabetes research [Internet]. 2016;2016:2129838–2129838. | No co-design |
| J. J, A.-M. H, A. L, D. K, S. R, L. T, et al. Effects of a physical activity and nutrition program in retirement villages: A cluster randomised controlled trial. The international journal of behavioral nutrition and physical activity [Internet]. 2017;14(1):92–92. | No co-design |
| B. W, N.S. C, J. B, M. C, A. N, R. M, et al. Early home-based pulmonary rehabilitation following acute exacerbation of COPD: A feasibility study using an action research approach. Chronic Respiratory Disease [Internet]. 2020;17 | No co-design |
| A.K.C. W, F.K.Y. W, Wong AKC, Wong FKY, Chang K. Effectiveness of a community-based self-care promoting program for community-dwelling older adults: a randomized controlled trial. Age and ageing [Internet]. 2019;48(6):852–8. | No co-design |
| D.W. S, M.A. W, K. A, M. P, H. S, G. T, et al. Effects of Home-Based Cardiac Rehabilitation on Time to Enrollment and Functional Status in Patients With Ischemic Heart Disease. Journal of the American Heart Association [Internet]. 2020;9(19):e016456–e016456. | No co-design |
| J. G, C. Y, S. K, E.V. E. EMPOWERING PATIENTS AND CARE PARTNERS TO TAKE CONTROL OF KIDNEY HEALTH: RESULTS OF A CKD PROBLEM-SOLVING INTERVENTION PILOT PROGRAM. American Journal of Kidney Diseases [Internet]. 2020;75(4):576–7. | No co-design |
| B. S, Stickney B, Vilshanskaya O. Engaging older people with English as a second language and frail older people in physical activity. Health promotion journal of Australia : official journal of Australian Association of Health Promotion Professionals [Internet]. 2005;16(2):116–23. | No co-design |
| Dondzila CJ, Perry CK, Bornstein DB. Enhancing Support for Physical Activity in Older Adults: A Public Health Call to Action. Journal of Public Health Management & Practice [Internet]. 2018;24(1):e26–9. | No co-design |
| BJ C, Esters J, MK C. Evaluation of the Revised Physical Activity Readiness Questionnaire in older adults. Medicine & Science in Sports & Exercise [Internet]. 1996;28(4):468–72. | No co-design |
| Abadi MH, Barker AM, Rao SR, Orner M, Rychener D, Bokhour BG, et al. Examining the Impact of a Peer-Led Group Program for Veteran Engagement and Well-Being. Journal of Alternative and Complementary Medicine [Internet]. 2021;27(S1):S37–44. | No co-design |
| D.R. B, M. de FNMAR, L.A. G, Y.A. de OD, Rodrigues Bueno D, de Fátima Nunes Marucci M, et al. Expenditures of medicine use in hypertensive/diabetic elderly and physical activity and engagement in walking: cross secctional analysis of SABE Survey. BMC geriatrics [Internet]. 2017;17(1):70–70. | No co-design |
| Hekler EB, Buman MP, Poothakandiyil N, Rivera DE, Dzierzewski JM, Morgan AA, et al. Exploring behavioral markers of long-term physical activity maintenance: a case study of system identification modeling within a behavioral intervention. Health education & behavior : the official publication of the Society for Public Health Education [Internet]. 2013;40(1 Suppl):51S-62S. | No co-design |
| Feng NC, Ryan E, Kidane M, Tusch ES, McFeeley BM, Carlsson R, et al. Feasibility of an at-home, web-based, interactive exercise program for older adults. Alzheimer’s and Dementia: Translational Research and Clinical Interventions [Internet]. 2019;5:825–33. | No co-design |
| V. C, H. M, C. V, S. S, L. T, F. M. Feasibility of an eight-week telerehabilitation intervention for patients with unresectable thoracic neoplasia receiving chemotherapy: A pilot study. Canadian Journal of Respiratory, Critical Care, and Sleep Medicine [Internet]. 2020;4(1):14–24. | No co-design |
| A. P, C.A. R, L. I, M. Z, J.R. O, Fried T.R.  AO  - Fried TR, et al. Feasibility of Delivering a Tailored Intervention for Advance Care Planning in Primary Care Practice. Journal of the American Geriatrics Society [Internet]. 2019;67(9):1917–21. | No co-design |
| Hardcastle SJ, Jimenez-Castuera R, Maxwell-Smith C, Bulsara MK, Hince D, S.J. H, et al. Fitbit wear-time and patterns of activity in cancer survivors throughout a physical activity intervention and follow-up: Exploratory analysis from a randomised controlled trial. PLoS ONE [Internet]. 2020;15(10):e0240967–e0240967. | No co-design |
| Collins K, Layne K, Schooley M, Chase L, Faradj-Bakht S, K. C, et al. Fitness in the Park: An Interprofessional Community-Based Partnership for Older Adults. Topics in Geriatric Rehabilitation [Internet]. 2021;37(3):186–90. | No co-design |
| B.A. T. For the love of it: Affective experiences that may increase physical activity participation among older adults. Social Science and Medicine [Internet]. 2016;161:61–3. | No co-design |
| Aoyagi Y, Park H, Park S, Shephard RJ, Y. A, H. P, et al. Habitual physical activity and health-related quality of life in older adults: interactions between the amount and intensity of activity (the Nakanojo Study). Quality of life research : an international journal of quality of life aspects of treatment, care and rehabilitation [Internet]. 2010;19(3):333–8. | No co-design |
| Aartolahti E, Hartikainen S, Lonnroos E, Hakkinen A, E. A, S. H, et al. Health and physical function predicting strength and balance training adoption: a community-based study among individuals aged 75 and older. Journal of aging and physical activity [Internet]. 2014;22(4):543–9. | No co-design |
| K.M. H, D.A. C, C.R. L, A. K, Heinrich KM, Crawford DA, et al. High-Intensity Functional Training Shows Promise for Improving Physical Functioning and Activity in Community-Dwelling Older Adults: A Pilot Study. Journal of geriatric physical therapy (2001) [Internet]. 2021;44(1):9–17. | No co-design |
| B.C. K, W.P. W, Remedios L.  AO  - Kwok BC, ORCID: http://orcid.org/0000-0002-4510-7907, Kwok BC, Wong WP, et al. Improving centre-based group exercise participation of older adults using the behaviour change wheel. BMJ open quality [Internet]. 2021;10(1). | No co-design |
| B.C. F, M.J. G, S.T. D, J. D, M. R, A.R. L, et al. Improving maintenance of physical activity in older, knee osteoarthritis patients trial-pilot (IMPACT-P): Design and methods. Contemporary Clinical Trials [Internet]. 2012;33(5):976–82. | No co-design |
| Garver MJ. Improving Maintenance of Physical ACtivity Trial (IMPACT-P) [Internet]. Improving Maintenance of Physical Activity Trial (Impact-p). Ohio State University; 2011. p. 233 p. | No co-design |
| A.V. C, M.M. D, S. S. Improving the cardio-respiratory fitness in older people and cardiac patients: Findings from an Italian participatory research. Monaldi Archives for Chest Disease [Internet]. 2003;60(2):107–10. | No co-design |
| A.M. C, C. M, S. C. Participation in community-based exercise maintenance programs after completion of hospital-based cardiac rehabilitation: A mixed method study. Journal of Cardiopulmonary Rehabilitation and Prevention [Internet]. 2011;31(1):42–6. | No co-design |
| Greenwood-Hickman MA, Rosenberg DE, Phelan EA, Fitzpatrick AL, M.A. GH, D.E. R, et al. Participation in Older Adult Physical Activity Programs and Risk for Falls Requiring Medical Care, Washington State, 2005-2011. Preventing chronic disease [Internet]. 2015;12:E90–E90. | No co-design |
| A. L, C.F. M, A. M, A.L. L, C.J. H, A.T. B, et al. Participation in Physical Activity During Center and Home-Based Pulmonary Rehabilitation for People With COPD: A SECONDARY ANALYSIS OF A RANDOMIZED CONTROLLED TRIAL. Journal of Cardiopulmonary Rehabilitation and Prevention [Internet]. 2019;39(2):E1–4. | No co-design |
| Annesi JJ, Westcott WL, J.J. A. Relations of physical self-concept and muscular strength with resistance exercise-induced feeling state scores in older women. Perceptual and Motor Skills [Internet]. 2007;104(1):183–90. | No co-design |
| Avioz-Sarig O, Olatunji S, Sarne-Fleischmann V, Edan Y. Robotic System for Physical Training of Older Adults. International journal of social robotics [Internet]. 2020;1–16. | No co-design |
| A.-L.T. HH, L. M, V.M. V, Huynh-Hohnbaum ALT, Marshall L, Villa VM, et al. Self-Management of Heart Disease in Older Adults. Home health care services quarterly [Internet]. 2015;34(3–4):159–72. | No co-design |
| B. S, C. S, L. P, G. T. Promoting physical activity engagement for people with multiple sclerosis living in rural settings: A proof-of-concept case study. European Journal of Physiotherapy [Internet]. 2017;19(Supplement 1):17–21. | No co-design |
| V. G, A.M. L, B. N, A. F. Pulmonary rehabilitation for moderate COPD (GOLD 2)-does it have an effect? COPD: Journal of Chronic Obstructive Pulmonary Disease [Internet]. 2011;8(5):380–6. | No co-design |
| D.A. G, R.U. N, A. G, S.J. L, A. S, C. M, et al. Randomized controlled trial of a peer led multimodal intervention for men with prostate cancer to increase exercise participation. Psycho-Oncology [Internet]. 2018;27(1):199–207. | No co-design |
| Benedetti TRB, Rech CR, Konrad LM, Almeida FA, Brito FA, Chodzko-Zajko W, et al. Re-thinking Physical Activity Programs for Older Brazilians and the Role of Public Health Centers: A Randomized Controlled Trial Using the RE-AIM Model. Frontiers in public health [Internet]. 2020;8:48–48. | No co-design |
| Anokye N, Mansfield L, Kay T, Sanghera S, Lewin A, Fox-Rushby J. The effectiveness and cost-effectiveness of a complex community sport intervention to increase physical activity: an interrupted time series design. BMJ open [Internet]. 2018;8(12):e024132–e024132. | No co-design |
| K. U, M. Y, Uemura K, Yamada M, Okamoto H. The Effectiveness of an Active Learning Program in Promoting a Healthy Lifestyle among Older Adults with Low Health Literacy: A Randomized Controlled Trial. Gerontology [Internet]. 2021;67(1):25–35. | No co-design |
| M.L.T. C, Chan MLT, Yu DSF. The effects of low-impact moderate-intensity stepping exercise on fatigue and other functional outcomes in older adults with multimorbidity: A randomized controlled trial. Archives of Gerontology and Geriatrics [Internet]. 2022;98:104577–104577. | No co-design |
| AM J, Rooks D, Lachman M, TH L, Levenson C, Heislein D, et al. Home-based resistance training: predictors of participation and adherence. Gerontologist [Internet]. 1998;38(4):412–21. | No co-design |
| Cohen GD, Perlstein S, Chapline J, Kelly J, Firth KM, Simmens S. Impact of professionally conducted cultural programs on the physical health, mental health, and social functioning of older adults--2-year results. Journal of Aging, Humanities, and the Arts [Internet]. 2007;1(1–2):5–22. | No co-design |
| A.L. M, J.R. B, C. ES, M. G. Longitudinal changes in physical function and physical activity in older adults. Age and Ageing [Internet]. 2018;47(4):558–64. | No co-design |
| Boulton ER, Horne M, Todd C, E.R. B, M. H, Todd C.  AO  - Boulton ER, et al. Multiple influences on participating in physical activity in older age: Developing a social ecological approach. Health expectations: an international journal of public participation in health care and health policy [Internet]. 2018;21(1):239–48. | No co-design |
| E. C, R.R. W, M. L, S. D, Cunningham E, Weaver RR, et al. Nordic Pole Walking for Individuals with Cancer: A Feasibility Randomized Controlled Trial Assessing Physical Function and Health-Related Quality of Life. Rehabilitation Oncology [Internet]. 2020;38(2):81–91. | No co-design |
| Arnett M, Toevs SE, Bond L, Hannah E. Outcomes of Participation in a Community-Based Physical Activity Program. Frontiers in public health [Internet]. 2019;7:225–225. | No co-design |
| ALPAY L, KOSTER Y. Persuasive eHealth to Support Home Rehabilitation of the Elderly After a Hip Operation: An Explorative Approach...30th Medical Informatics Europe Conference. Studies in Health Technology & Informatics [Internet]. 2020;270:1349–50. | No co-design |
| Andrews RM, Tan EJ, Varma VR, Rebok GW, Romani WA, Seeman TE, et al. Positive Aging Expectations Are Associated With Physical Activity Among Urban-Dwelling Older Adults. The Gerontologist [Internet]. 2017;57(suppl_2):S178–86. | No co-design |
| Johnson L, Deatrick EJ, Oriel K. The Use of Music to Improve Exercise Participation in People with Dementia: A Pilot Study. Physical & Occupational Therapy in Geriatrics [Internet]. 2012;30(2):102–8. | No co-design |
| Fleig L, McAllister MM, Chen P, Iverson J, Milne K, McKay HA, et al. Health behaviour change theory meets falls prevention: Feasibility of a habit-based balance and strength exercise intervention for older adults. Psychology of Sport & Exercise [Internet]. 2016;22:114–22. | No co-design |
| Feldman MB, Arakaki LS, Raker AR. Participation in a Self-management Intervention for People Living With HIV. The Journal of the Association of Nurses in AIDS Care : JANAC [Internet]. 2016;27(4):530–7. | No co-design |
| Cheng WLS. The effects of mutual goal-setting practice in older adults with chronic illness. Geriatric nursing (New York, NY) [Internet]. 2018;39(2):143–50. | No co-design |
| Bjaras G, Harberg LK, Sydhoff J, Ostenson CG. Walking campaign: a model for developing participation in physical activity? Experiences from three campaign periods of the Stockholm Diabetes Prevention Program (SDPP). Patient education and counseling [Internet]. 2001;42(1):9–14. | No co-design |
| A. C, J.S. L, G. K, V. A, S. S, N. H. The value of a post-polio syndrome self-management programme. Journal of Thoracic Disease [Internet]. 2020;12:S153–62. | No co-design |
| Razai MS, Oakeshott P. The Importance of Community and Patient Involvement in the Design of Physical Activity Programs. Journal of Primary Care & Community Health [Internet]. 2020;11:1–1. | No co-design |
| Menkin JA, McCreath HE, Song SY, Carrillo CA, Reyes CE, Trejo L, et al. “Worth the Walk”: Culturally Tailored Stroke Risk Factor Reduction Intervention in Community Senior Centers. Journal of the American Heart Association [Internet]. 2019;8(6):e011088–e011088. | No co-design |
| B.C. F, K.A. C, N.E. R, Farmer BC, Croteau KA, Richeson NE, et al. Using pedometers as a strategy to increase the daily steps of older adults with chronic illness: from research to practice. Home healthcare nurse [Internet]. 2006;24(7):449–56. | No co-design |
| Chester H, Beresford R, Clarkson P, Entwistle C, Gillan V, Hughes J, et al. The Dementia Early Stage Cognitive Aids New Trial (DESCANT) intervention: A goal attainment scaling approach to promote self-management. International Journal of Geriatric Psychiatry [Internet]. 2021;36(5):784–93. | No co-design |
| C.F. L, N. C, R.M.E. L, D.A. M, Leask CF, Colledge N, et al. Co-Creating Recommendations to Redesign and Promote Strength and Balance Service Provision. International journal of environmental research and public health [Internet]. 2019;16(17):3169–3169. | No comparison group |
| Timmerman JG, Tonis TM, Dekker-van Weering MGH, Stuiver MM, Wouters MWJM, van Harten WH, et al. Co-creation of an ICT-supported cancer rehabilitation application for resected lung cancer survivors: design and evaluation. BMC health services research [Internet]. 2016;16:155–155. | No comparison group |
| Singleton A, Raeside R, Partridge SR, Hayes M, Maka K, Hyun KK, et al. Co-designing a Lifestyle-Focused Text Message Intervention for Women After Breast Cancer Treatment: Mixed Methods Study. Journal of Medical Internet Research [Internet]. 2021;23(6):N.PAG-N.PAG. | No comparison group |
| M.M. G, C. M, A.M. H, F. S, Green MM, Meyer C, et al. Co-designing Being Your Best program-A holistic approach to frailty in older community dwelling Australians. Health & social care in the community [Internet]. 2021; | No comparison group |
| A. B, P.I. O, G.E. F. A prospective study examining the influence of cardiac rehabilitation on the sedentary time of highly sedentary, physically inactive patients. Annals of Physical and Rehabilitation Medicine [Internet]. 2018;61(4):207–14. | No comparison group |
| Aberg AC, Halvorsen K, From I, Bruhn AB, Oestreicher L, Melander-Wikman A, et al. A Study Protocol for Applying User Participation and Co-Learning Lessons Learned from the eBalance Project. International journal of environmental research and public health [Internet]. 2017;14(5):512–512. | No comparison group |
| Da Silva Junior JLA, Biduski D, Bellei EA, Becker OHC, Daroit L, Pasqualotti A, et al. A Bowling Exergame to Improve Functional Capacity in Older Adults: Co-Design, Development, and Testing to Compare the Progress of Playing Alone Versus Playing With Peers. JMIR serious games [Internet]. 2021;9(1):e23423– e23423. | No comparison group |
| Munoz J, Mehrabi S, Li Y, Basharat A, Middleton LE, Cao S, et al. Immersive Virtual Reality Exergames for Persons Living With Dementia: User-Centered Design Study as a Multistakeholder Team During the COVID-19 Pandemic. JMIR serious games [Internet]. 2022;10(1):e29987–e29987. | No comparison group |
| H. M, L. N, A. B, C. H, S.M. G, E. L, et al. Implementation of a co-designed physical activity program for older adults: positive impact when delivered at scale. BMC public health [Internet]. 2018;18(1):1289–1289. | No comparison group |
| Wilcox S, Laken M, Bopp M, Gethers O, Huang P, McClorin L, et al. Increasing physical activity among church members: community-based participatory research. American journal of preventive medicine [Internet]. 2007;32(2):131–8. | No comparison group |
| Stevens AB, Thiel SB, Thorud JL, Smith ML, Howell D, Cargill J, et al. Increasing the Availability of Physical Activity Programs for Older Adults: Lessons Learned From Texercise Stakeholders. Journal of aging and physical activity [Internet]. 2016;24(1):39–44. | No comparison group |
| Swallow D, Petrie H, Power C, Lewis A, Edwards ADN, D. S, et al. Involving Older Adults in the Technology Design Process: A Case Study on Mobility and Wellbeing in the Built Environment. Studies in health technology and informatics [Internet]. 2016;229:615–23. | No comparison group |
| Travers J, Romero-Ortuno R, Ni She E, Cooney MT, J. T, R. RO, et al. Involving older people in co-designing an intervention to reverse frailty and build resilience. Family practice [Internet]. 2022;39(1):200–6. | No comparison group |
| S.L. S, R.K. W, J.H. L, L. F, A. Z, L.N. G, et al. Development of an Exergame for Urban-dwelling Older Adults With Functional Limitations: Results and Lessons Learned. Progress in community health partnerships: research, education, and action [Internet]. 2016;10(1):73–81. | No comparison group |
| Hong YA, Goldberg D, Ory MG, Towne SDJ, Forjuoh SN, Kellstedt D, et al. Efficacy of a Mobile-Enabled Web App (iCanFit) in Promoting Physical Activity Among Older Cancer Survivors: A Pilot Study. JMIR cancer [Internet]. 2015;1(1):e7–e7. | No comparison group |
| Lee JLC, Ho RTH, J.L.C. L, Ho R.T.H.  AO  - Lee  Rainbow Tin Hung, ORCID: http://orcid.org/0000-0002-6173-621X JLC, ORCID: http://orcid.org/0000- 0001-8447-6855 A O - Ho. Engaging community-dwelling older adults as co-developers in a public outdoor exercise facilities-based physical activity education intervention: A mixed-method participatory study in Hong Kong. Health & social care in the community [Internet]. 2021; | No comparison group |
| A. A, G. C, R. B. Enhancing uptake of an exercise programme after stroke or transient ischaemic attack (TIA)-a co-production event. International Journal of Stroke [Internet]. 2018;13(3 Supplement 1):29–29. | No comparison group |
| Masterson-Algar P, Williams S, Burton CR, Arthur CA, Hoare Z, Morrison V, et al. Getting back to life after stroke: co-designing a peer-led coaching intervention to enable stroke survivors to rebuild a meaningful life after stroke. Disability & Rehabilitation [Internet]. 2020;42(10):1359–72. | No comparison group |
| Verhoeven F, Cremers A, Schoone M, van Dijk J. Mobiles for mobility: Participatory design of a “Happy walker” that stimulates mobility among older people. Gerontechnology [Internet]. 2016;15(1):32–44. | No comparison group |
| B. P, R. J, M. W, L. LO. Older adults’ experiences of behavior change support in a digital fall prevention exercise program: Qualitative study framed by the self determination theory. Journal of Medical Internet Research [Internet]. 2021;23(7):e26235–e26235. | No comparison group |
| Anderson AS, Craigie AM, Gallant S, McAdam C, Macaskill EJ, McKell J, et al. Optimisation of the ActWELL lifestyle intervention programme for women attending routine NHS breast screening clinics. Trials [Internet]. 2020;21(1):484–484. | No comparison group |
| Davies J, Lester C, O’Neill M, Williams G. Sustainable participation in regular exercise amongst older people: Developing an action research approach. Health Education Journal [Internet]. 2008;67(1):45–55. | No comparison group |
| Chen EK, Reid MC, Parker SJ, Pillemer K. Tailoring evidence-based interventions for new populations: a method for program adaptation through community engagement. Evaluation & the health professions [Internet]. 2013;36(1):73–92. | No comparison group |
| Bammann K, Recke C, Albrecht BM, Stalling I, Doerwald F, K. B, et al. Promoting Physical Activity Among Older Adults Using Community-Based Participatory Research With an Adapted PRECEDE-PROCEED Model Approach: The AEQUIPA/OUTDOOR ACTIVE Project. American journal of health promotion : AJHP [Internet]. 2021;35(3):409–20. | No comparison group |
| D.M.J. W, M. K, V. C, R. B, J. C, P. Z, et al. The development and codesign of the PATHway intervention: a theory-driven eHealth platform for the self management of cardiovascular disease. Translational behavioral medicine [Internet]. 2019;9(1):76–98. | No comparison group |
| Apps LD, Mitchell KE, Harrison SL, Sewell L, Williams JE, Young HM, et al. The development and pilot testing of the self-management programme of activity, coping and education for chronic obstructive pulmonary disease (SPACE for COPD). International journal of chronic obstructive pulmonary disease [Internet]. 2013;8:317–27. | No comparison group |
| Brox E, Konstantinidis ST, Evertsen G. User-Centered Design of Serious Games for Older Adults Following 3 Years of Experience With Exergames for Seniors: A Study Design. JMIR serious games [Internet]. 2017;5(1):e2–e2. | No comparison group |
| D. M, A. I, M. D, G. J, J. L, J. E, et al. Using co-creation and multi-criteria decision analysis to close service gaps for underserved populations. Health expectations: an international journal of public participation in health care and health policy [Internet]. 2019;22(5):1058–68. | No comparison group |
| Barnard S, Dunn S, Reddic E, Rhodes K, Russell J, Tuitt TS, et al. Wellness in Tillery: a community-built program. Family and Community Health [Internet]. 2004;27(2):151–7. | No comparison group |
| Ummels D, Braun S, Stevens A, Beekman E, Beurskens A. Measure It Super Simple (MISS) activity tracker: (re)design of a user-friendly interface and evaluation of experiences in daily life. Disability and Rehabilitation: Assistive Technology [Internet]. 2020 Sep 24 [cited 2022 Jun 15];1–11. | No comparison group |
| Schmidt F, Ribi K, Haslbeck J, Urech C, Holm K, Eicher M. Adapting a peer-led self-management program for breast cancer survivors in Switzerland using a co-creative approach. Patient education and counseling [Internet]. 2020;103(9):1780–9. | No comparison group |
| Van der Weegen S, Verwey R, Spreeuwenberg M, et al. The development of a mobile monitoring and feedback tool to stimulate physical activity of people with a chronic disease in primary care: a user-centered design. *JMIR Mhealth Uhealth*. 2013;1(2):e8. Published 2013 Jul 2. doi:10.2196/mhealth.2526 | No comparison group |
| M.L. de F, L.P. L, B.A. de S. Balance, functional mobility and quality of life in elderly participants and non-participants of a community center. Scientia Medica [Internet]. 2017;27(4):ID27400–ID27400. | Not in english |
| E.J. L, H.H. H, M.W. H, N. MH, B. C, K.E. F, et al. Enhanced Medical Rehabilitation Increases Therapy Intensity and Engagement and Improves Functional Outcomes in Postacute Rehabilitation of Older Adults: A Randomized-Controlled Trial. Journal of the American Medical Directors Association [Internet]. 2012;13(8):708–12. | Institutionalized |
| Harris MA. Beat the Street: A Pilot Evaluation of a Community-Wide Gamification-Based Physical Activity Intervention. Games for health journal [Internet]. 2018;7(3):208–12. | Unable to access full text |
| Bertelsen P, Kenstrup A, M, Madsen J. Steps Toward Technology Design to Beat Health Inequality -- Participatory Design Walks in a Neighbourhood with High Health Risks. Studies in Health Technology & Informatics [Internet]. 2017;233:158–72. | Unable to access full-text |
