# Supplementary material for: The effects of co-designed physical activity interventions in older adults: A systematic review and meta-analysis
Source: PLoS One. 2024 May 10;19(5):e0297675. doi: 10.1371/journal.pone.0297675 (PMC11086838; doi:10.1371/journal.pone.0297675)
Supplement: S2 Table — (DOCX) [file pone.0297675.s004.docx]

S2 Table. Characteristics of included studies.

| **Study, Country, Design** | **Aim** | **Setting** | **Inclusion/Exclusion Criteria** | **Duration, Frequency, Time** | **Type** | **Providers** | **Co-design Details** | **Other Outcome Measures** |
| --- | --- | --- | --- | --- | --- | --- | --- | --- |
| Alma (2012), Netherlands, Pretest-  Posttest study | To increase participation | Two low-vision rehabilitation centres, two rural locations, and a home-based exercise component | IC: 1) visually impaired persons, 2) age ≥55y, 3) able to speak Dutch, 4) able to understand instructions, 5) referral to a low-vision rehabilitation centre; EC: NR | IG: 20-week program (1/week) + booster session at 12 weeks post, 2 hours per session | IG: Training of practical skills; education, social interaction and counseling and training of problem-solving skills; individual and group goal setting; and a home-based physical exercise programme  SC: Pre-test Group | IG: OT and SW | IG: Three focus group interviews with visually impaired elderly and delegates of interest groups along with an expert meeting with health professionals involved in rehabilitation informed the development and design. Program manual was written in consultation with the PT, OT, and SW from the low-vision rehabilitation centre | Autonomy (IPA questionnaire) |
| Brach (2017) USA, Cluster RCT | To improve function, disability, and walking ability | 32 independent living facilities, senior apartment buildings and senior community centres in the Greater Pittsburgh and Pennsylvania area | IC: 1) ≥65 years old, 2) a resident of the independent living facilities or apartment building, or member of the senior community center, 3) ambulate independently (with or without a straight cane) for household distances, and 4) have a usual gait speed of at least 0.60 m/s; EC: 1) are non-English speaking, 2) have impaired cognition, defined as unable to follow a 2-step command or understand the informed consent process, 3) plan to leave the area for an extended period of time over the ensuing 4 months, 4) have a progressive neuromuscular disorder, 5) have any acute medical condition or illness that is not stable, or 6) have an inappropriate physiologic response to the 6 minute walk test | 12-week program (2/week), 50 minutes per session | IG: A warm-up and cool down (range of motion and stretches for the lower extremities and trunk), stepping patterns, walking patterns, strengthening exercises focused on lower extremity muscle groups important for walking  SC: A warm-up and cool down (gentle range-of-motion exercises and stretches), upper and lower extremity strength exercises (used playground balls, the opposite extremity, and body weight), aerobic activities (marching, tapping, and skiing at various speeds). | Trained exercise leaders or trained staff activity personnel (i.e., PT/PTA) | IG: Participant stakeholders were involved in the pilot studies and focus groups to help develop the intervention and as members of the Community Advisory Boards for the execution and translation of the intervention | Participant satisfaction (exit surveys)  Adherence (attendance) |
| Chewning (2019), USA, RCT | To reduce falls risk | Three aging units and aging and disability resource centers | IC: 1) ≥ 65 years, 2) dwelling independently in the community, 3) agreeing to be randomized, 4) reporting a fall in the last year or having a strong fear of falling; EC: 1) using a walker indoors, 2) having a terminal illness, 3) being hospitalized or in a nursing home in the past 2 months, 4) anticipated absence from two tai chi sessions and receiving physical therapy or a community course or balance exercise program for falls prevention in the previous 2 months | IG: 6-week program (2/week), 90 minutes per session | IG: Tai chi Warm-ups and Basic Moves instruction; exercise break and socialization; Participants designed individual weekly tai chi practice plans with coaching; Tai Chi Fundamental Short Form instruction; Qi Gong closing; and Traditional Mind Body Skills Training. Educated to use Fab Four tai chi movements in everyday life.  Participants practiced at home.  SC: Wait list (same intervention after study) | IG: Trained instructors | IG: Stakeholder groups including community site coordinators from each center in the study, older adults representative of study participants, and course instructors regularly informed key study decisions about recruitment strategies and materials, sustainable program implementation, participants’ home practice planners and trackers, data collection, and refinement of course manuals for dissemination | Balance confidence (ABC Scale)  Executive function (Trail Making Test Part B) |
| Estabrooks (2005), USA, RCT | To increase moderate intensity PA | USDHHS Elderly Nutrition Program congregate meal sites operated out of local community centers | IC: 1) “regular” meal site attendees from one of the four selected sites, 2) must have attended ≥ one day per week over the month before recruitment; EC: NR | IG: 12-week program (1/week), 45 minutes per session + 3 supportive telephone calls, 10-15 minutes per telephone call  SC: 6 presentations (2/week), 45 minutes per session | IG: Group based social cognitive intervention and supportive telephone calls to assess current health status, review recent PA, and discuss goals  SC: Education on various topics on PA. The program was developed by the local cooperative extension system | Trained research assistants | Collaborated with Administration on Aging in a large Midwestern state and congregate meal-site users in the area to inform study design, research questions, and progression | Group cohesion (PAGE-Q)  Self efficacy (self report scale) |
| Gong (2015), China, Longitudinal cluster RCT | To encourage PA | From 12 community health centers in 6 urban districts | IC: 1) ≥ 55 years of age, 2) diagnosed with hypertension by a doctor or a physician, following the standard procedure, 3) currently on antihypertensive medication, 4) be able to attend intervention research activities, including lectures, group-meetings, and telephone counseling, and 5) can safely participate in moderate or higher levels of physical activities; EC: 1) failing the Physical Activity Readiness Questionnaire test; 2) unable to complete the research study due to physical, and mental abnormalities; 3) suffering from medical conditions that may interfere with participating in intervention and/or engaging in required levels of physical activities; and 4) electrocardiogram evidence of heart dysfunction | IG: 6-week program (1/week) + two booster sessions at 3 month post, Session I 45-60 minutes, II 45-60 minutes, III 10-20 minutes, IV 45-60 minutes, V 10-20 minutes, VI 45-60 minutes, VII 10-20 minutes, VIII 45-60 minutes | IG: Two group lectures, two sessions of one-on-one telephone counseling, and two group meetings focused on addressing PA barriers, improving self-efficacy, increasing self-motivation, and empowering participants to increase their PA levels. After the 3-month follow-up assessment, a two-session booster was arranged starting with telephone-counseling followed by a group meeting. The IG also received the SC  SC: Periodic monitoring of blood pressure and hypertension related cardiovascular and other complications, medication adherence, general physical checkup, and psychological counseling | Trained interventionists (physicians) | IG: A program was developed by a group of experts in public health and health education, physicians and nurses, community leaders, retired officials and a number of hypertensive patient volunteers and their family members. The program was circulated among the key investigators of the study for feedback and revision to produce a pilot version | Blood pressure (mmHg)  PA (transition stage toward PA and levels of PA self report scale) |
| Kittipimpanon (2012), Thailand, Pretest-Posttest study | To prevent falls | One community in the catchment area of Ramathibodi Hospital | IC: 1) ≥ 60 years of age 2) living in the selected study site community 3) being able to verbally communicate in Thai 4) being willing to participate 5) demonstrating adequate cognitive function; EC: NR | IG: 12-week program (2/week) + fall education program 1/year + home visits 2/year, 45 minutes per session | IG: Group fall education focused on risk factors and prevention strategies. Teaching methods included: discussion; sharing experiences; relationship building between elders; and distribution of printed fall prevention material. Balance/exercise group activity taught and performed. Participants were encouraged to perform the balance/exercise activity daily by giving handouts for home. Home visits to review medication and discuss potential environmental changes  SC: Pre-test Group | IG: A public health nurse delivered the education and trained team members instructed group exercise sessions | IG: Three elders and six stakeholders (one public health nurse; two public health volunteers; elder club president; and two community leaders) were involved in the situational analysis phase, a workshop on fall prevention, and developed the fall program. Five organizations (Public Health Center; District Office; Property Bureau; local temple; and, local market) were identified that would support the fall prevention program | Demographics and fall history (DDFHQ)  Fall risk (Thai-FRAT)  Fall prevention(FPBQ)  Environment (EHAQ)  Physical strength and function (PPT)  Partnerships (PC)  Satisfaction (SFPPQ) |
| Perry (2011), USA, Pretest-  Posttest study | To increase PA and social interaction | Community gym in an urban neighbourhood in Seattle, Washington | IC: 1) older adult, 2) aged 60-85, 3) clearance from primary healthcare provider; EC: 1) age < 60 or > 85, 2) no medical clearance | IG: 8-week program (1/week), 60 minute per session | IG: Tai Chi program with youth and older adult pairs who performed interactive exercises or poses  SC: Pre-test Group | IG: Experienced tai chi instructor | IG: Community advisory board met regularly for 6 months to develop the program followed by a community advisory board member admin survey and focus groups with older adults and youths’ parents for intergenerational PA program interest and program preferences | PA enjoyment (PACES)  Feasibility (attendance, interviews, focus groups) |
| Sieno (2021), Japan, A 2-year cluster non RCT | To develop a frailty prevention strategy | 18 districts in Ota City, Japan within the community | IC: 1) 65 yrs or older, 2) living in Ota City; EC: Those with long-term care insurance certification and those admitted to hospitals or residing in nursing homes | IG: 2-year education and exercise program | IG: Resident-oriented activities related to frailty prevention (physical exercise, nutrition, and/or social participation) and improving the community environment through patient education  SC: Usual health practices. | NR | IG: Various professionals including residents, welfare professionals, exercise instructors, “community organizations”, companies, research institutions, and government employees discussed and developed the intervention based on a baseline survey and community consultations. | Physical activity (weekly exercise frequency, walking duration)  Physical function (motor fitness scale, mobility limitation)  Dietary variety (DVS, FFS)  Psychosocial function (self-report questionnaire)  Awareness |
| Abbreviations: ALIVE = Active Living in Vulnerable Elders, KM2H2 = Keep Moving toward Healthy Heart and Healthy Brain, NR = not reported, IG = intervention group, SC = standard care, USA = United States of America, USDHHS = United States Department of Health and Human Services, PA = physical activity, DDFHQ = Data and Fall History Questionnaire, Thai - FRAT = Thai Fall Risk Assessment Test, FPBQ = Fall Prevention Behaviors Questionnaire, EHAQ = Environment Hazard Assessment Questionnaire, PPT = Physical Performance Test, PC = Partnership Checklist, SFPPQ = Satisfaction with Fall Prevention Program Questionnaire, PACES = Physical Activity Enjoyment Scale, IC = inclusion criteria, EC = exclusion criteria, MA = mean age, NA = not applicable, OT = occupational therapist, SW = social worker, RCT = randomized controlled trial, PT = physiotherapist, PTA = physiotherapist assistant, PAGE-Q = Physical Activity Group Environment Questionnaire, DVS = dietary variety score, FFS = food frequency score | | | | | | | | |
