# Supplementary material for: The effects of co-designed physical activity interventions in older adults: A systematic review and meta-analysis
Source: PLoS One. 2024 May 10;19(5):e0297675. doi: 10.1371/journal.pone.0297675 (PMC11086838; doi:10.1371/journal.pone.0297675)
Supplement: S1 Fig — (PDF) [file pone.0297675.s005.pdf]

S1 Figure. Risk of bias summaries.

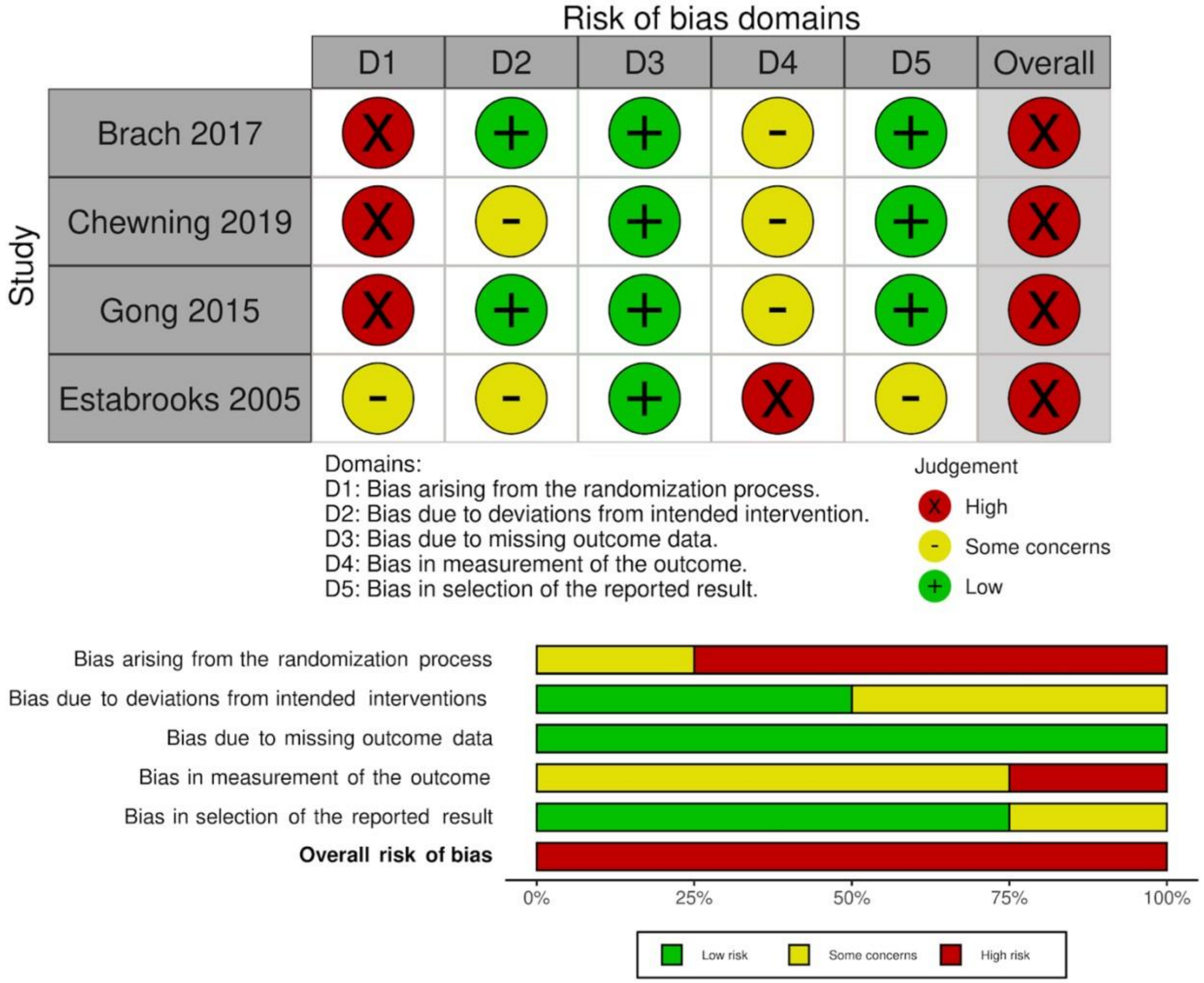

Fig 1. Cochrane risk of bias summary for each included randomized controlled trial for physical activity outcomes.

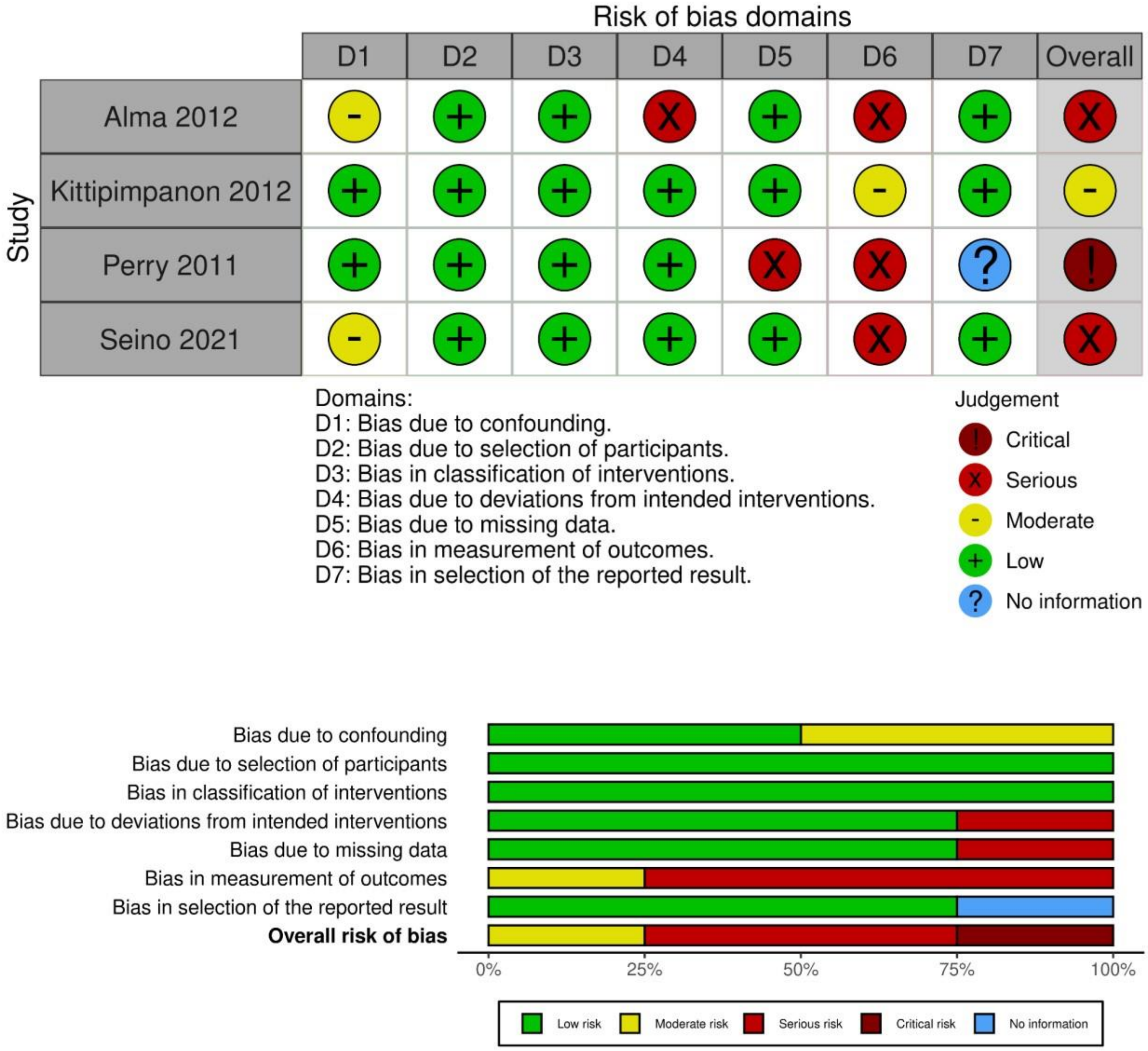

Fig 2. ROBINS-I risk of bias summary for each included non-randomized controlled trial for physical activity outcomes.
