# Supplementary figures and images for: The effects of co-designed physical activity interventions in older adults: A systematic review and meta-analysis
Source: PLoS One. 2024 May 10;19(5):e0297675. doi: 10.1371/journal.pone.0297675 (PMC11086838; doi:10.1371/journal.pone.0297675)

S2 Figure. GRADE summary.
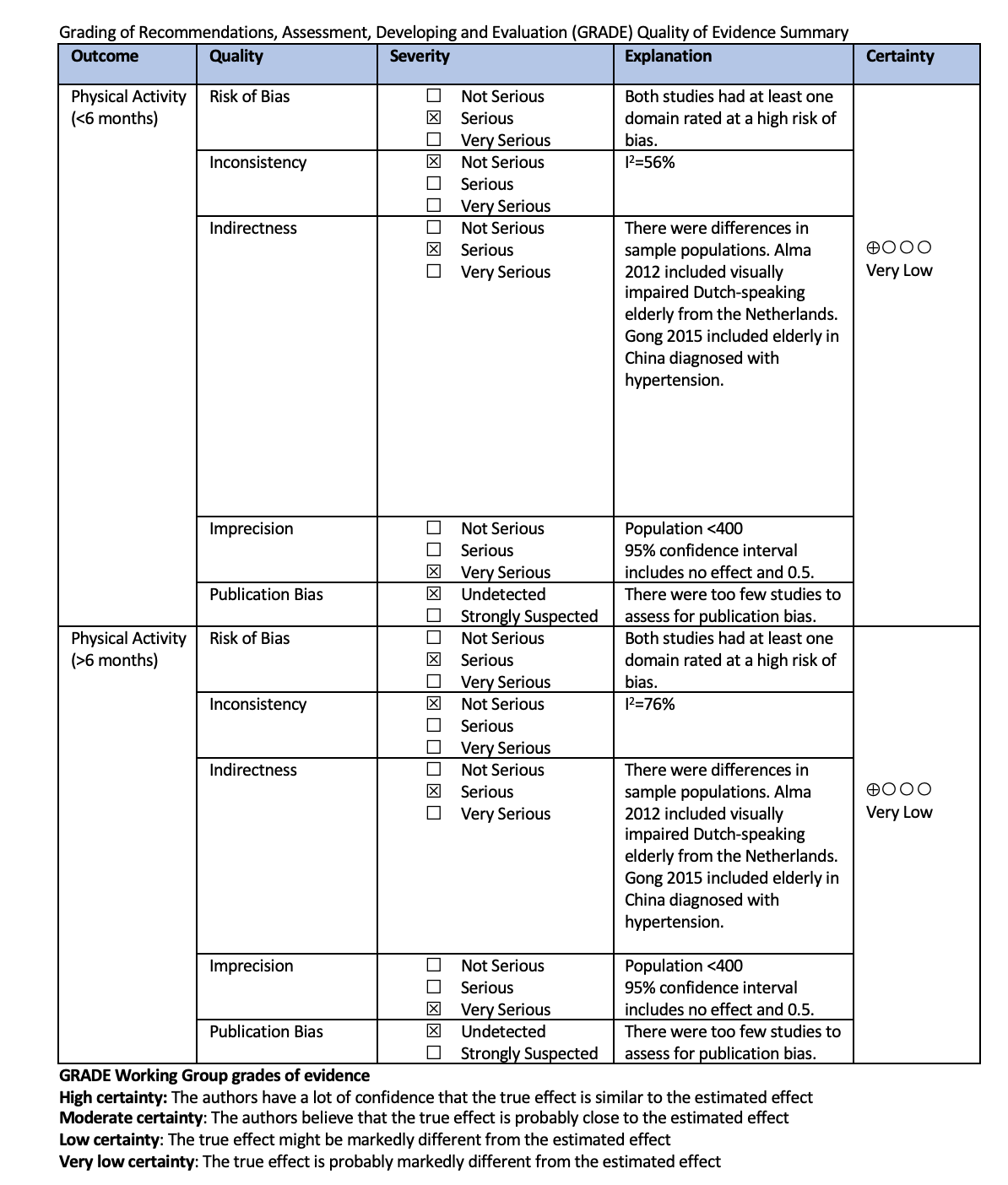

Supplement: S2 Fig — (DOCX) [file pone.0297675.s006.docx]
